# Trehalose‐6‐phosphate phosphatase E modulates ABA‐controlled root growth and stomatal movement in *Arabidopsis*

**DOI:** 10.1111/jipb.12925

**Published:** 2020-04-16

**Authors:** Wenjing Wang, Qingbin Chen, Shouming Xu, Wen‐Cheng Liu, Xiaohong Zhu, Chun‐Peng Song

**Affiliations:** ^1^ State Key Laboratory of Crop Stress Adaptation and Improvement, School of Life Sciences Henan University Kaifeng 475001 China; ^2^ Department of Biology and Food Science Shangqiu Normal University Shangqiu 476000 China

**Keywords:** ABA, ROS, root elongation, stomatal movement, trehalose

## Abstract

Trehalose plays important roles in plant growth and stress responses and is synthesized from trehalose‐6‐phosphate by trehalose‐6‐phosphate phosphatase (TPP). Here, we show that trehalose and abscisic acid (ABA) have synergistic effects on root growth and stomatal closure. The *Arabidopsis thaliana* genome contains ten genes encoding TPPs and the expression level of one, *TPPE*, and trehalose contents increased in response to ABA. In the presence of ABA, the ABA‐responsive transcription factor ABA RESPONSE ELEMENT BINDING FACTOR2 (ABF2) directly binds to the *TPPE* promoter to activate its expression. Genetic analysis revealed that *TPPE* acts downstream of *ABF2*, which is supported by the findings that *TPPE* expression and trehalose content are reduced in the *abf2* mutant and that a mutation in *TPPE* abolished the ABA‐sensitive root elongation phenotype of *35S:ABF2* plants. Reactive oxygen species (ROS) accumulation in response to ABA failed to occur in *tppe* mutant plants, suggesting that *TPPE* is involved in ABA‐controlled root elongation and stomatal movement by inducing ROS accumulation. This study uncovers a new branch of the ABA signaling pathway and provides a molecular basis for the role of trehalose in plant responses to abiotic stress.

## Graphical abstract

Trehalose and ABA are known for their roles in plant stress response. In this study, we demonstrate a synergistic effect of trehalose and ABA on root elongation and stomatal closure. Trehalose‐6‐phosphate phosphatase E (TPPE) controls trehalose accumulation and modulates ABA‐controlled root growth and stomatal movement in Arabidopsis.

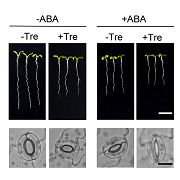

## INTRODUCTION

The non‐reducing disaccharide trehalose is composed of two glucose molecules linked by α,α‐1,1‐glycoside bond. In bacteria and yeast, trehalose plays roles in resistance to environmental stresses such as dehydration and heat (Hounsa et al. 1998). In insects, trehalose serves as a major source of sugar, participating in growth, development, molting, and metamorphosis (Elbein et al. 2003). In plants, trehalose plays an important role in regulating gene expression, thus tuning plant metabolism, growth, development, and stress responses (Paul et al. 2008). Expressing a bacterial or yeast trehalose biosynthesis gene to alter trehalose content significantly affected abiotic stress tolerance in tobacco (*Nicotiana tabacum*), *Arabidopsis thaliana*, rice (*Oryza sativa*), potato (*Solanum tuberosum*), and other plants (Miranda et al. 2007; Kondrak et al. 2011; Kondrak et al. 2012).

In eukaryotes, trehalose‐6‐phosphate synthase (TPS) catalyzes the transfer of glucose from UDP‐glucose to glucose‐6‐phosphate (G6P) to produce trehalose‐6‐phosphate (T6P), which is dephosphorylated by trehalose‐6‐phosphate phosphatase (TPP) to produce trehalose (Avonce et al. 2006). Trehalose‐6‐phosphate, the precursor of trehalose, inhibits the activity of the protein kinase SnRK1 and is involved in regulating plant respiration, starch synthesis, and sucrose metabolism (O'Hara et al. 2013; Figueroa 2016; Paul and Gonzalez‐Uriarte 2018). The *A. thaliana* genome contains 11 *TPS* genes (*TPS1–TPS11*). The absence of *TPS1* leads to an embryo lethal phenotype (Ramon et al. 2009; Vandesteene et al. 2010). In addition, germination and stomatal movement of *tps1* mutants are hypersensitive to abscisic acid (ABA) compared to the wild type (WT) (Gomez et al. 2010). Overexpressing *OsTPS1* in rice seedlings enhanced tolerance to a variety of abiotic stresses by increasing trehalose levels and upregulating the expression of several abiotic stress‐related genes (Li et al. 2011; Yang et al. 2012; Han et al. 2016).

By contrast, the roles of TPPs in trehalose production, as well as their biological functions, are poorly understood. Analysis of ten *Arabidopsis TPPs* (*TPPA–TPPJ*) revealed that these genes exhibit different tissue‐specific expression patterns, pointing to their possible functional diversity (Vandesteene et al. 2012; Van Houtte et al. 2013). However, there is some overlap in their expression patterns, suggesting that their functions might be partially redundant. Moreover, phylogenetic analysis clustered each TPP and related TPPs from other species into separate groups (Ma et al. 2007; Li et al. 2008). *TPP* expression is induced by different hormone and abiotic stress treatments and TPPs function in plant stress responses. For example, AtTPPF plays an active role in protecting cells from reactive oxygen species (ROS) damage by increasing the levels of soluble sugars in plants under drought stress (Lin et al. 2019). *AtTPPD* overexpression lines exhibited significantly enhanced salt tolerance due to hypersensitivity to redox changes in two cysteine residues of TPPD. The activity increased rapidly under salt stress, which led to trehalose accumulation (Krasensky et al. 2014). Overexpressing *OsTPS1* and *OsTPP1* in rice increased trehalose contents and improved plant survival under low‐temperature stress (Ge et al. 2008). However, the specific mechanism underlying the activities of other TPPs remains to be explored.

Abscisic acid regulates many processes in plants, including seed germination and dormancy, stomatal movement, and adaptation to stress (Cutler et al. 2010). In *Arabidopsis*, the ABA signaling pathway is mediated by three types of proteins: regulatory component of ABA receptor (RCAR)/pyrabactin resistance (PYR)/PYR1‐like proteins (PYLs), protein phosphatase 2Cs (PP2Cs), and sucrose non‐fermenting‐1‐related protein kinase 2s (SnRK2s). In the presence of ABA, SnRK2s are activated, leading to the phosphorylation‐mediated activation or repression of downstream components such as transcription factors and membrane channel proteins (Fujii et al. 2009; Fujita et al. 2013). Most ABA‐induced genes contain a conserved ABA‐responsive *cis*‐element (ABRE; PyAC GTGG/TC) in their promoter regions. The basic leucine zipper (bZIP) transcription factor ABRE binding protein (AREB) binds to the ABRE motif and activates the expression of ABA‐responsive genes (Choi et al. 2000; Yoshida et al. 2010; Fujita et al. 2013; Wang et al. 2019b). *Trehalose‐6‐phosphate phosphatases* also participate in the ABA signaling pathway. For example, *tppb*, *tppg*, and *tppf* mutants show altered sensitivity to ABA during seed germination and stomatal opening (Vandesteene et al. 2012). However, the detailed molecular mechanism underlying the roles of *TPPs* in the ABA signaling pathway remains largely unknown.

In the current study, we examined the effects of exogenous trehalose and ABA treatment on root elongation and stomatal closure in *Arabidopsis*. TPPE is encoded by the most highly ABA‐responsive *TPP* family gene in *Arabidopsis* and we demonstrate that TPPE regulates trehalose accumulation. Furthermore, we show that ABF2 is an upstream transcription factor that mediates ABA‐regulated *TPPE* expression. Our results reveal the molecular basis of ABA‐regulated trehalose metabolism in *Arabidopsis.*

## RESULTS

### Trehalose and ABA act synergistically on root elongation and stomatal closure

**Figure 1:**
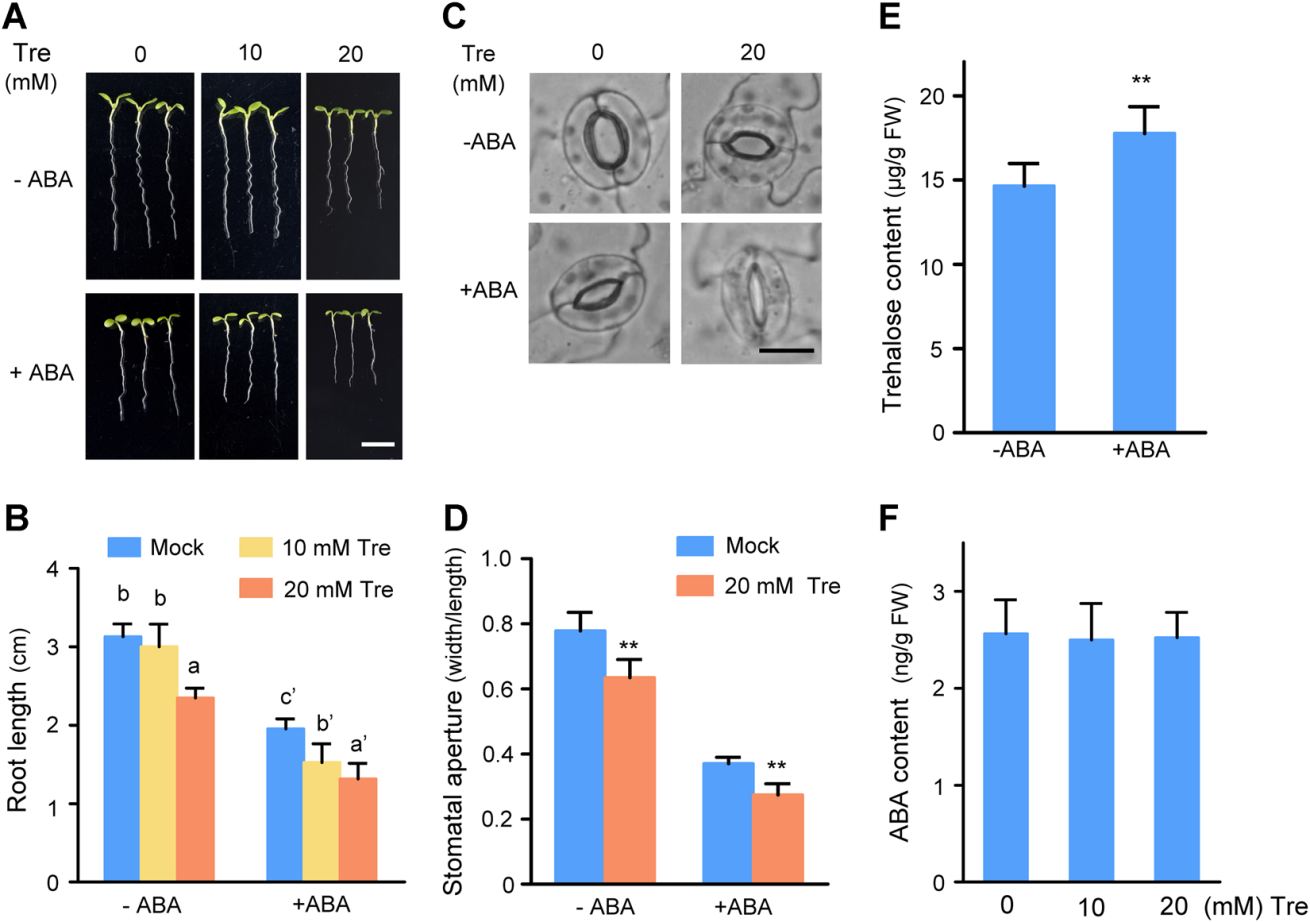
**Exogenous application of trehalose enhances the effects of abscisic acid (ABA) on root elongation and stomatal closure in *Arabidopsis*** **(A)** Photographs of root elongation in wild type (WT) seedlings grown on ½ MS plates containing 0 μM (−ABA) or 30 µM ABA (+ABA) with different concentrations of trehalose (0, 10, and 20 mM). Scale bar, 1 cm. **(B)** Statistical analysis of root length of the plants shown in **(A)**. The values are means ± *SD* (*n* > 10). Different letters indicate significant differences at *P* < 0.05 (one‐way analysis of variance (ANOVA)). **(C)** Stomatal morphology in plants under trehalose and ABA treatment. Scale bar, 10 μm. **(D)** Statistical analysis of stomatal aperture after trehalose and ABA treatment. Error bars indicate ± *SD* (*n* > 100). Significant differences are based on Student's *t*‐test: *P < 0.05, **P < 0.01. **(E)** Trehalose content of WT seedlings in the presence of ABA. Values are means ± *SD* (*n* = 3). The asterisk indicates a significant difference compared to the control using Student's *t*‐test (*P < 0.05, **P < 0.01). **(F)** ABA contents in seedlings under exogenous trehalose treatment. Values are means ± SD (*n* = 3).

Exogenous trehalose application causes stomatal closure and inhibits root elongation in *Arabidopsis* (Shi et al. 2019). To determine the role of trehalose in the ABA pathway, we analyzed the effects of trehalose and ABA on root elongation and stomatal aperture. We transferred 4‐d‐old WT seedlings to half‐strength Murashige and Skoog (½ MS) medium with or without 30 μM ABA combined with various concentrations of trehalose. The root length was significantly shorter in seedlings grown on medium supplemented with 10 mM trehalose and 30 μM ABA than in seedlings grown in the presence of 30 μM ABA alone. Root growth was more severely reduced when the seedlings were treated with 20 mM trehalose and 30 μM ABA (Figure 1A,B). To examine the effects of trehalose and ABA on stomatal closure, we added trehalose and ABA to stomatal opening solution. The presence of exogenous trehalose enhanced the effect of ABA‐induced stomatal closure (Figure 1C,D). These results indicate that trehalose and ABA have synergistic effects on root elongation and stomatal closure.

We also examined the trehalose content of plants treated with exogenous ABA and vice versa. ABA treatment induced trehalose accumulation *in vivo* (Figure 1E), whereas trehalose treatment had no effect on ABA levels (Figure 1F). The expression of *ABSCISIC ACID DEFICIENT2* (*ABA2*) and *NINE‐CIS‐EPOXYCAROTENOID DIOXYGENASE3* (*NCED3*), key genes in the ABA biosynthesis pathway (Iuchi et al. 2001; Endo et al. 2008), did not significantly differ in the presence versus absence of trehalose (Figure S1). These results indicate that trehalose does not affect the ABA biosynthesis pathway, and they suggest that trehalose functions downstream of ABA signaling during root elongation and stomatal closure.

### Abscisic acid regulates trehalose content through *TPPE*

To further explore the molecular network by which trehalose regulates ABA‐mediated root elongation and stomatal closure, we analyzed the expression of *TPP* gene family members in response to ABA. Based the *Arabidopsis* eFP browser (https://bar.utoronto.ca/efp/cgi‐bin/efpWeb.cgi), the expression levels of *TPPE*, *TPPF*, and *TPPI* increase significantly after ABA treatment. To verify this, we used quantitative reverse transcription polymerase chain reaction (qRT‐PCR) to measure the expression levels of *TPPs* in 10‐d‐old WT seedlings before and after ABA treatment. Abscisic acid treatment induced *TPPE* expression to levels much higher than those of the other *TPPs*, with *TPPE* reaching its highest value (7.5‐fold increase) at 3 h after ABA treatment (Figure S2). In addition, we generated reporter lines harboring *GUS* driven by the *TPPE* native promoter and performed histochemical staining of seedlings after ABA treatment (Figure S3). GUS activity dramatically increased in the presence of ABA, suggesting that ABA upregulates the expression of *TPPE.*

*TPPE* complements the function of the yeast *tps2* mutant (Vogel et al. 1998). To identify the function of *TPPE* in *Arabidopsis*, we examined the catalytic activity of this enzyme expressed in *Escherichia coli*. TPPE had higher catalytic activity for T6P than for glucose‐6‐phosphate (G6P) and sorbitol‐6‐phosphate (S6P) *in vitro* (Figure S4), indicating that T6P is a specific substrate of TPPE.

### *TPPE* participates in ABA‐inhibited root elongation and stomatal movement

To gain a better understanding of the role of *TPPE* in trehalose production and ABA responses, we measured the trehalose contents in *tppe* mutants and *TPPE* overexpression lines. Two independent T‐DNA insertion mutants of *TPPE* were identified: *tppe‐1* (SALK_090223), with a T‐DNA insertion in an intron, and *tppe‐2* (GK‐291G05), with a T‐DNA insertion in the coding sequence (Figure S5A, B). *TPPE* expression was significantly lower in *tppe‐1* and *tppe‐2* and higher in the *35S:TPPE* lines compared to the WT, as determined by qRT‐PCR (Figure S5C, D).

**Figure 2:**
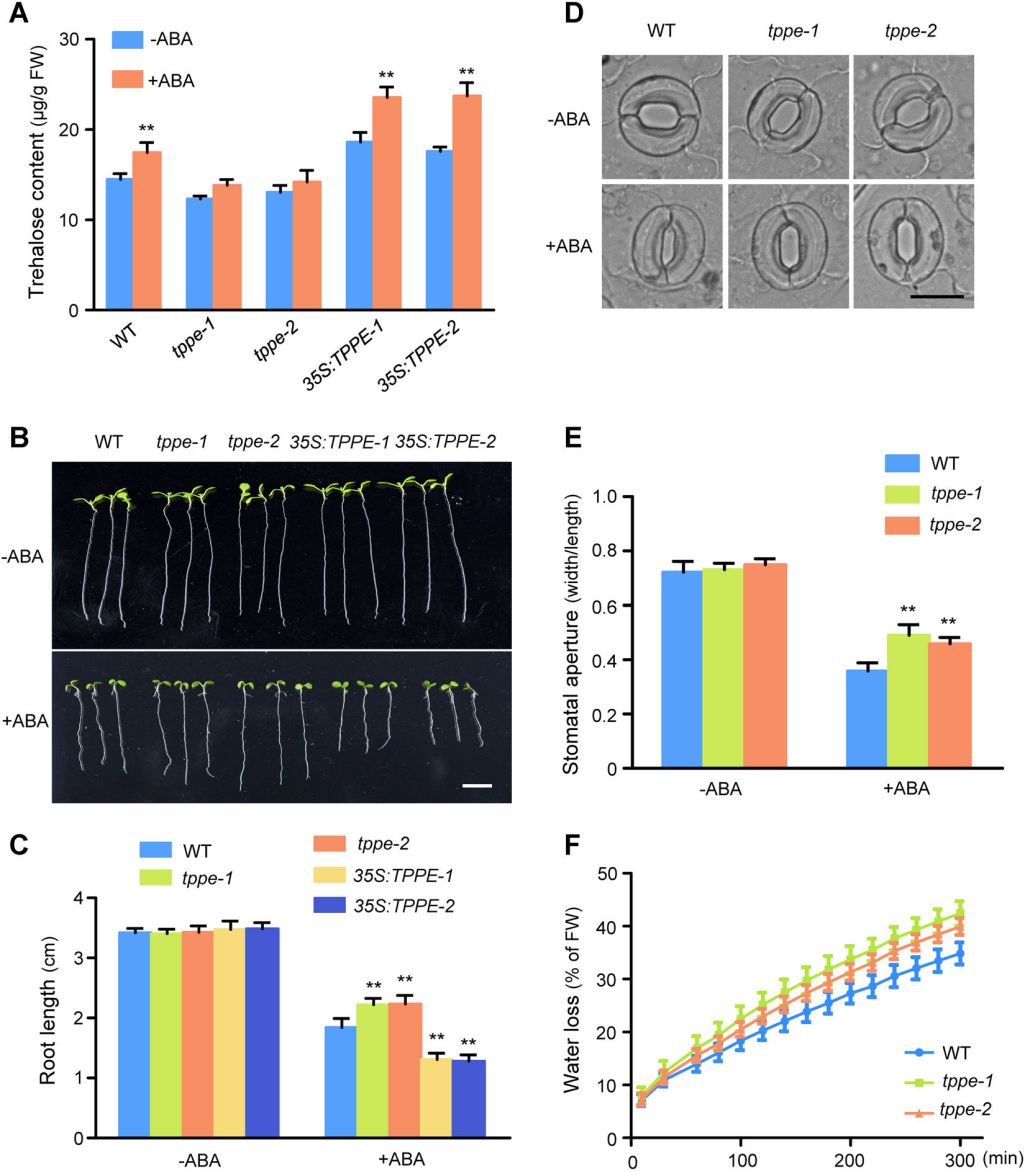
**Loss‐of‐function mutants of *TPPE* are less sensitive to abscisic acid (ABA) than the wild type (WT) in terms of root inhibition and stomatal closure** **(A)** Trehalose contents in 10‐d‐old WT, *tppe‐1*, *tppe‐2*, and *TPPE* overexpression plants with or without ABA treatment (50 μM). The values are means ± *SD* (*n* > 10). *P < 0.05, **P < 0.01 (Student's *t*‐test). **(B)** Comparison of root elongation among genotypes on ½ MS with or without 30 μM ABA. Scale bar, 1 cm. **(C)** Statistical analysis of the differences in root length among the plants shown in **(B)** The values are means ± SD (*n* > 10), *P < 0.05, **P < 0.01 (Student's *t*‐test). **(D)** Representative images showing stomatal apertures before and after 2 h treatment with 10 μM ABA. Scale bar, 10 μm. **(E)** Quantification of stomatal apertures. The values are means ± SD (*n* > 100) from five biological replicates. *P < 0.05, **P < 0.01 (Student's *t‐*test). **(F)** The detached leaves of 4‐week‐old WT, *tppe‐1*, and *tppe‐2* plants were used for relative water loss measurements. The experiments were repeated three times with similar results. Each data point represents the mean ± SD (*n* = 3).

We then analyzed the trehalose content in 10‐d‐old seedlings of these genotypes by LC‐MS. The trehalose content was higher in *35S:TPPE* and lower in *tppe‐1* and *tppe‐2* compared to the WT (Figure 2A). In addition, we measured trehalose contents in the plants in response to the application of 50 μM ABA. The trehalose content increased (1.21‐fold) in the WT after ABA treatment but remained almost unchanged in *tppe‐1* and *tppe‐2* after this treatment (1.09 and 1.08‐fold, respectively). By contrast, the trehalose content of *35S:TPPE* increased 1.26‐fold under ABA treatment. Collectively, these results indicate that ABA induces *TPPE* expression and positively regulates trehalose accumulation.

To investigate the role of *TPPE* in the ABA response, we analyzed the sensitivity of root growth to ABA in the WT, *TPPE* overexpression lines, and *tppe* mutants. We transferred 4‐d‐old seedlings grown on ½ MS medium to medium supplemented with 30 μM ABA. After 5 d of growth, root growth was dramatically retarded in the overexpression lines under ABA treatment compared to the WT, whereas root elongation was less sensitive to ABA in *tppe* seedlings (Figure 2B,C).

We measured stomatal aperture in WT and *tppe* plants to determine whether *TPPE* regulates ABA‐dependent stomatal closure. In the light, the stomatal apertures of *tppe* and WT plants were similar (Figure 2D). However, stomatal closure in the *tppe‐1* and *tppe‐2* mutants was significantly less sensitive to ABA than the WT (Figure 2E). Water loss measured in detached leaves reflects the stomatal aperture of intact leaves. Consistent with the stomatal closure results, water loss in detached leaves occurred much more rapidly in *tppe‐1* and *tppe‐2* than in the WT; the water loss rates were approximately 7.6% and 5.2% faster in *tppe‐1* and *tppe‐2* versus the WT, respectively, at 300 min after detachment (Figure 2F). However, no significant difference was observed in leaf water loss between the overexpression lines and the WT (data not shown). The *tppe* deletion mutant lines, which were generated using the CRISPR/Cas9 system, showed the same phenotype as the *tppe‐1* and *tppe‐2* T‐DNA insertion mutants (Figure S6). Taken together, these data demonstrate that TPPE positively regulates ABA‐mediated root‐growth inhibition and stomatal closure.

### ABA RESPONSE ELEMENT BINDING FACTOR2 directly binds to the *TPPE* promoter *in vitro* and *in vivo*

**Figure 3:**
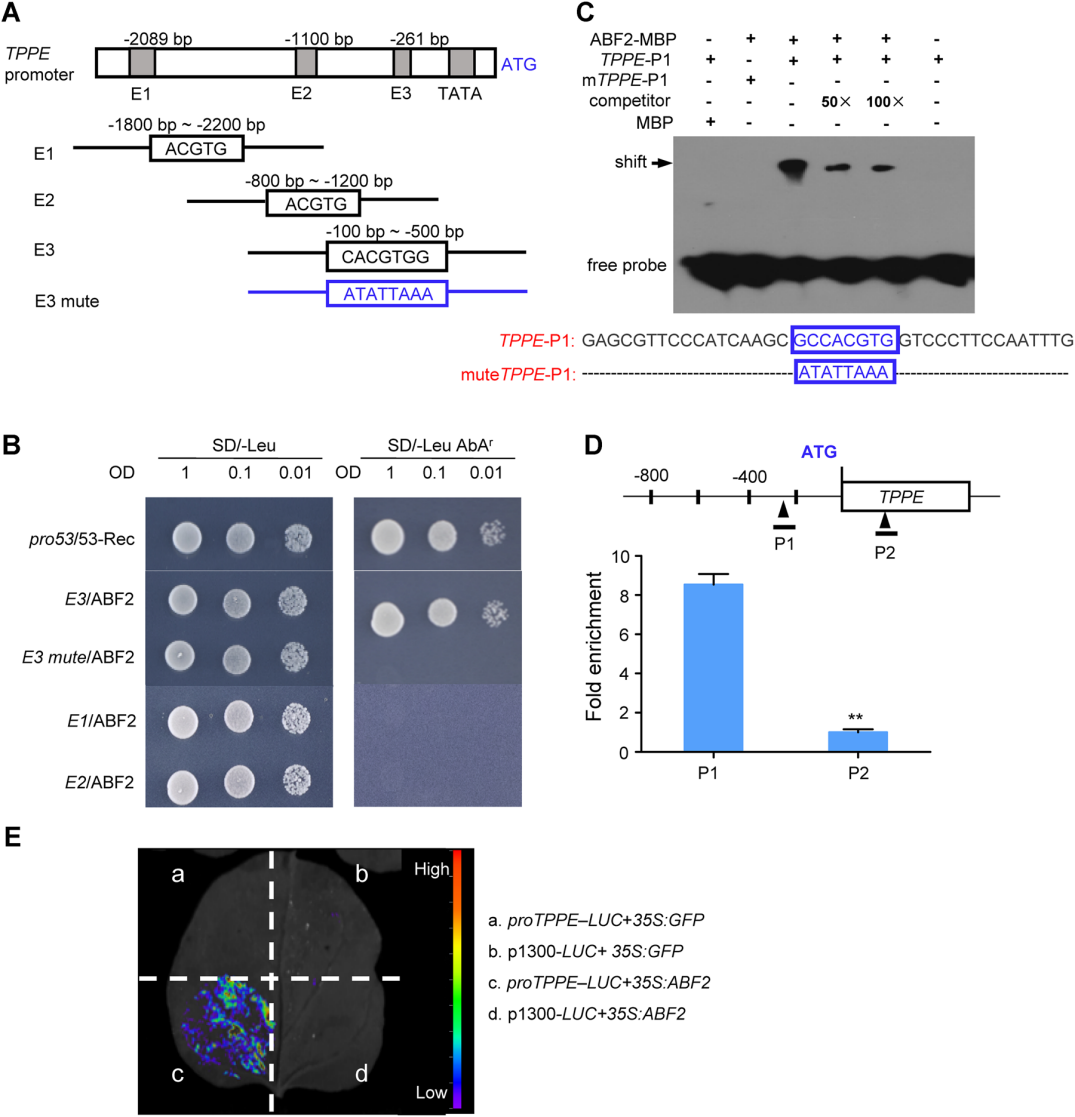
**ABA RESPONSE ELEMENT BINDING FACTOR2 (ABF2) binds to *TPPE in vivo* and *in vitro*** **(A)** Sequence analysis of the *TPPE* promoter. The gray boxes upstream of ATG represent the TATA box and three ABA‐responsive *cis*‐elements (ABREs). Each ABRE was selected as the core region, and the line indicates the truncated promoter region. **(B)** Physical interactions of ABF2 with the *TPPE* promoter in yeast one‐hybrid (Y1H) assays. **(C)** Electrophoretic mobility shift assay (EMSA) of *in vitro* binding of ABF2 to the ABRE motif of the *TPPE* promoter. A probe (mute *TPPE‐*P1) with the G‐box/ABRE motif mutated was used to test binding specificity. “−” represents the absence and “+” represents the presence of components in the reaction. Sequences of both the wild‐type and mutated probes are shown under the images, with the G‐box/ABRE motif boxed. **(D)** chromatin immunoprecipitation polymerase chain reaction (ChIP‐PCR) assay of *in vivo* binding of ABF2‐GFP to the *TPPE* promoter. Two DNA fragments (P1, P2) spanning these sites were examined by ChIP enrichment tests, as shown in the schematic diagram. Fold enrichment was calculated as the ratio of *35S:ABF2‐GFP* to *35S:GFP* signal. Data are means ± *SD* of three repeats, *P < 0.05, **P < 0.01. **(E)** Transcriptional activity assay in *N. benthamiana* leaves. ABF2 promotes the expression of the reporter gene *LUC* driven by the ABRE‐containing promoter.

To explore the mechanisms by which ABA regulates *TPPE* expression, we identified transcription factors that directly regulate the transcription of *TPPE* and predicted the ABREs in the promoter region of *TPPE* using PlantCARE (http://bioinformatics.psb.ugent.be/webtools/plantcare/html/). Three ABREs in the *TPPE* promoter were predicted to bind to AREB transcription factors. Using these fragments as bait in yeast one‐hybrid assays (Figure 3A), we identified the bZIP transcription factor ABF2 as binding the *TPPE* promoter E3 region. We next used full‐length coding region of ABF2 to test if ABF2 binding to the *TPPE* promoter region could induce gene expression. Indeed, co‐expression with *ABF2* induced the expression of the aureobasidin A resistance reporter gene (*AbA^r^*) driven by the *E3* fragment of the *TPPE* promoter region (pAbAi‐*E3*). By contrast, yeast co‐transformed with pAbAi‐*E1*, pAbAi‐*E2*, and ABF2 exhibited abnormal growth (Figure 3B).

We then performed an electrophoretic mobility shift assay (EMSA) to determine whether ABF2 directly binds to the *TPPE* promoter. A 40 bp biotin‐labeled DNA fragment containing the ABRE consensus motif (CTCGTGG), was used as a probe (*TPPE*‐P1), and a DNA fragment harboring a mutated ABRE was used as a control (m*TPPE*‐P1). We carried out a competition assay by adding 50‐fold and 100‐fold excess amounts of unlabeled probe. When labeled probes were pre‐incubated with the ABF2‐MBP protein, a shifted band was detected (Figure 3C). By contrast, the binding of the ABF2‐MBP protein to the promoter was abolished by the mutation in m*TPPE*‐P1, as well as by the addition of excess unlabeled probe. These results suggest that ABF2 physically interacts with the *TPPE* promoter *in vitro*.

To determine whether ABF2 directly binds to the promoter of *TPPE in vivo*, we performed a chromatin immunoprecipitation (ChIP) assay using *ABF2:GFP*‐overexpressing plants. Chromatin isolated from *ABF2:GFP* transgenic plants and *35S:GFP* control plants was immunoprecipitated with GFP antibody, followed by quantitative polymerase chain reaction (qPCR) to quantify the fold enrichment of the *TPPE* promoter regions. We observed an 8.4‐fold enrichment of the *TPPE* promoter region containing the ABRE, but no enrichment in the coding region of *TPPE* (Figure 3D). These results indicate that ABF2 directly binds to the ABRE‐containing region of the *TPPE* promoter *in vivo*.

Next, we performed an *in vivo* luciferase activation assay to further explore the binding of ABF2 to the *TPPE* promoter. The *TPPE* promoter region, that is, a 1.0 kb fragment upstream of the ATG, was fused to the firefly luciferase reporter gene (*LUC*), transferred into *Agrobacterium* GV3101, and transiently transformed into *Nicotiana benthamiana* leaves. Luminescence was detected only in the area co‐transformed with *35S:ABF2* and *ProTPPE‐LUC* (Figure 3E). These results indicate that ABF2 binds to the *TPPE* promoter to activate *LUC* expression in *N. benthamiana* leaves. Taken together, these results suggest that ABF2 binds to the promoter of *TPPE in vitro* and *in vivo.*

### ABA RESPONSE ELEMENT BINDING FACTOR2 controls *TPPE* expression and affects trehalose production

**Figure 4:**
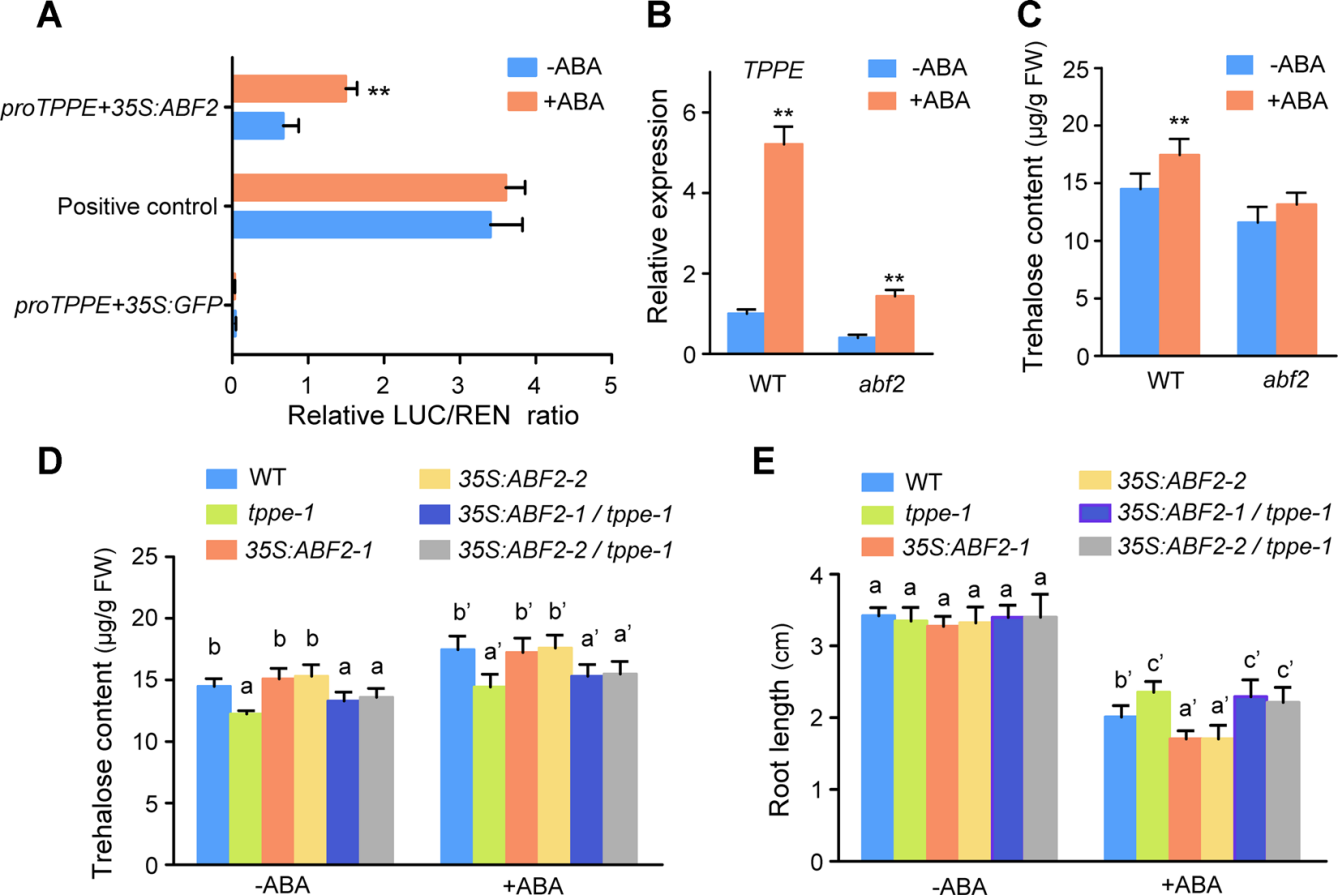
**ABA RESPONSE ELEMENT BINDING FACTOR2 (ABF2) mediates abscisic acid (ABA)‐controlled trehalose accumulation and root growth inhibition** **(A)** Transactivation of the *TPPE* promoter by ABF2 in protoplasts. Dual‐luciferase assays were carried out to test ABA‐induced binding of ABF2 to the *TPPE* promoter in *Arabidopsis* protoplasts. The empty effector construct *35S:GFP* was used as a control. Relative luciferase activity was determined 18 h after transfection with or without ABA treatment. Data are means ± *SD* (Student's *t*‐test, *n* > 3, *P < 0.05, **P < 0.01). **(B)** Expression levels of *TPPE* in wild type (WT) and *abf2* plants after ABA treatment. Induction levels of *TPPE* in 10‐d‐old plants by ABA (50 μM, 4 h) were determined by qRT‐PCR. Values are mean ± *SD* (Student's *t*‐test, *n* > 3, *P < 0.05, **P < 0.01). **(C)** Trehalose contents of WT and *abf2* plants with or without ABA treatment. **(D)** Trehalose contents in 10‐d‐old *tppe‐1*, *35S:ABF2*, *35S:ABF2/tppe‐1*, and WT plants with or without ABA treatment. The values are means ± *SD* (*n* > 3). Different letters indicate significant differences at *P* < 0.05 (one‐way analysis of variance (ANOVA)). **(E)** Statistical analysis of root length in plants under ABA treatment. *tppe‐1*, *35S:ABF2*, *35S:ABF2/tppe‐1*, and WT seedlings were incubated on ½ MS or ½ MS with ABA for 5 d. Root length was measured, which was repeated at least three times in each experiment. The values are means ± *SD* (*n* > 10). Different letters indicate significant differences at *P* < 0.05 (one‐way ANOVA).

ABA RESPONSE ELEMENT BINDING FACTOR2 is an important transcription factor that bridges ABA signaling and plays key roles in plant responses to environmental stress. To examine whether ABA regulates the expression of *TPPE* through ABF2, we performed a dual‐luciferase reporter assay using *Arabidopsis* protoplasts. The ABRE motif between −290 bp and −230 bp upstream of the ATG codon of *TPPE* was fused to the *LUC* reporter vector in the form of four repeats and co‐transformed with the effector vector into WT leaf protoplasts with or without ABA treatment. The transient overexpression of ABF2 activated the expression of the reporter gene, and the LUC/REN ratio sharply increased (2.25‐fold) after 10 μM ABA treatment (Figure 4A). These results indicate that ABA induces the expression of *TPPE* through ABF2.

To explore whether *ABF2* functions upstream of *TPPE* in ABA‐induced trehalose accumulation in *Arabidopsis*, we obtained the T‐DNA insertion mutant *abf2* (SALK_002984) (Figure S7A, B). The *abf2* and *tppe* mutants exhibited similar root elongation patterns when grown on ABA‐containing medium (Figure S7D), implying that *ABF2* and *TPPE* act in the same signaling pathway. We examined the expression of *TPPE* in *abf2* via qRT‐PCR. *TPPE* was expressed at significantly lower levels in *abf2* versus the WT (Figure 4B). The expression of *TPPE* in the mutant increased after 50 μM ABA treatment, but it was still significantly lower than that in the WT. The trehalose content in *abf2* did not significantly change in response to ABA treatment (Figure 4C). These results indicate that ABF2 is required for ABA‐induced increases in *TPPE* expression and trehalose content *in vivo*.

To investigate whether ABF2 participates in the regulation of trehalose‐modulated root elongation under ABA treatment, we examined whether the *tppe‐1* mutation would suppress the phenotypes of *ABF2*‐overexpressing plants under ABA treatment. We generated *35S:ABF2/tppe‐1* plants by crossing the *35S:ABF2* overexpression line with *tppe‐1* (Figure S7E). The trehalose content in *35S:ABF2/tppe‐1* was similar to that in *tppe‐1* (Figure 4D). In addition, the seedling growth of the *35S:ABF2/tppe‐1* was less sensitive to ABA compared to the *35S:ABF2* line (Figure 4E)*.* These results indicate that ABF2 is a positive regulator of *TPPE* that functions in ABA‐mediated root elongation.

### TPPE participates in ABA‐induced ROS accumulation

**Figure 5:**
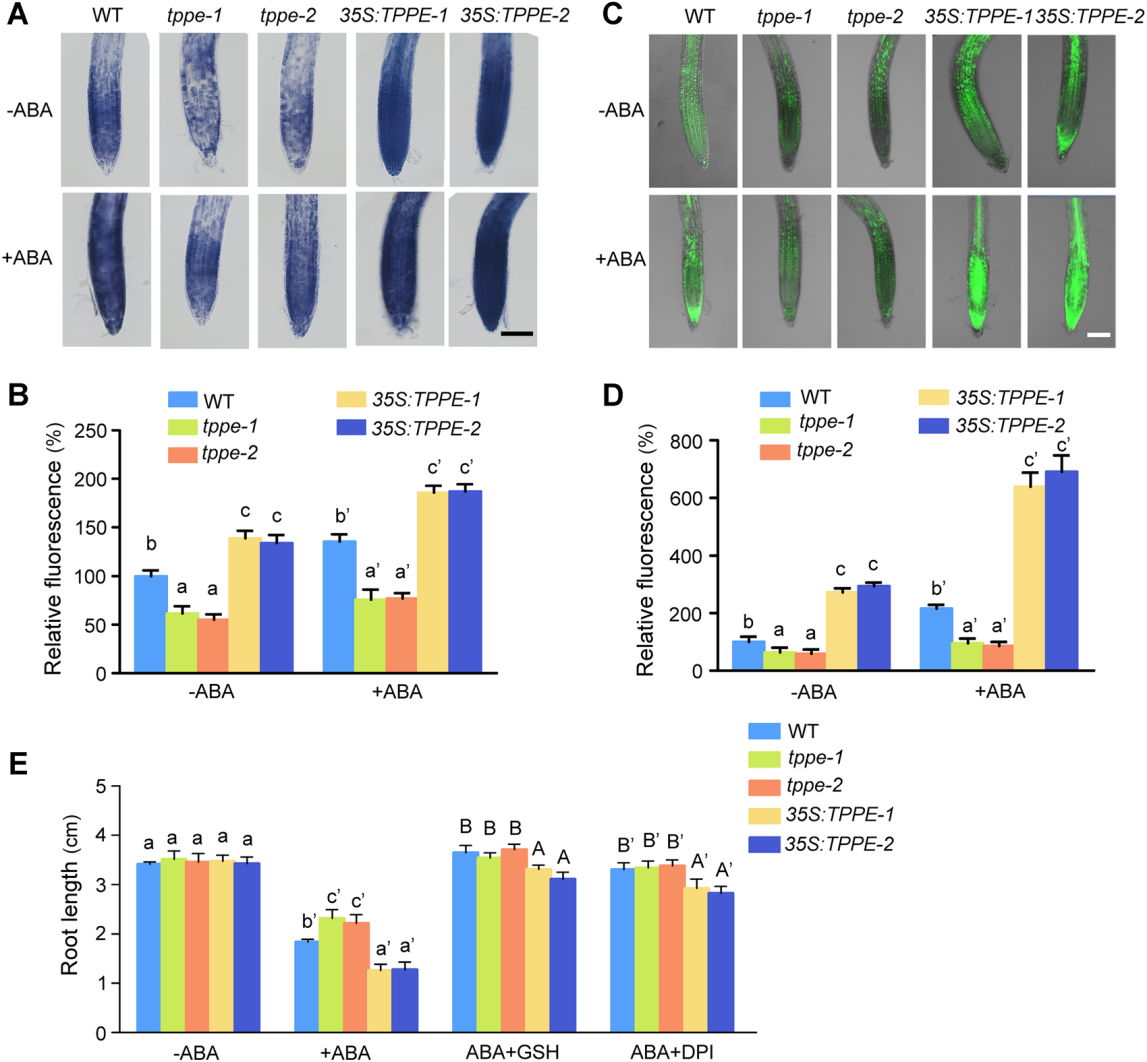
**Trehalose enhances abscisic acid (ABA)‐induced reactive oxygen species (ROS) accumulation in roots and stomata** **(A)** Nitroblue tetrazolium chloride (NBT) staining for superoxide anion in the primary roots of wild type (WT), *tppe‐1*, *tppe‐2*, *35S:TPPE‐1*, and *35S:TPPE‐2* seedlings after ABA treatment. Scale bar, 100 μm. **(B)** Relative intensities calculated from **(A)**. The values are means ± *SD* (*n* > 20). Different letters indicate significant differences at *P* < 0.05 (one‐way analysis of variance (ANOVA)). **(C)** H_2_DCFDA staining for ROS in primary roots of WT, *tppe‐1*, *tppe‐2*, *35S:TPPE‐1*, and *35S:TPPE‐2* seedlings after ABA treatment. Scale bar, 100 μm. **(D)** Relative intensities calculated from **(C)**. The values are means ± *SD* (*n* > 10). Different letters indicate significant differences at *P* < 0.05 (one‐way ANOVA). **(E)** Statistical analysis of the root lengths of seedlings grown on ½ MS medium supplemented with ABA and 100 µM GSH or 2 µM DPI. The values are means ± *SD* (*n* > 10). Different letters indicate significant differences at *P* < 0.05 (one‐way ANOVA).

Trehalose promotes ROS production, which can also be elicited by ABA treatment (Yang et al. 2014; Shi et al. 2019). Therefore, we investigated whether TPPE modulates the ABA‐induced accumulation of ROS. Hydrogen peroxide (as revealed by H_2_DCFDA staining) and superoxide (as revealed by nitroblue tetrazolium chloride (NBT) staining) were detected in the root tips of WT, *tppe‐1*, *tppe‐2*, *35S:TPPE‐1*, and *35S:TPPE‐2* seedlings. In the absence of ABA, less H_2_O_2_ and superoxide accumulated in the root tips of *tppe‐1* and *tppe‐2* than in the WT, whereas the highest levels of ROS were detected in the root tips of *35S:TPPE‐1* and *35S:TPPE‐2* seedlings. However, ABA‐induced ROS production was partially impaired in *tppe‐1* and *tppe‐2*, whereas *35S:TPPE* exhibited enhanced ROS levels compared to WT (Figure 5). Furthermore, we analyzed ROS accumulation and stomatal movement in these plants in response to ABA using the fluorogenic reagent H_2_DCFDA. The fluorescence intensity of guard cells was lower in *tppe‐1* and *tppe‐2* versus WT plants after ABA treatment (Figure S8). These results indicate that decreased trehalose levels impair ABA‐induced ROS production and that *TPPE* participates in ABA‐induced ROS accumulation. Therefore, TPPE is involved in ABA‐induced ROS accumulation in root tips and guard cells.

Finally, to verify the notion that TPPE participates in ABA‐induced ROS accumulation to inhibit root elongation, we transferred 4‐d‐old WT, *tppe‐1*, *tppe‐2*, *35:TPPE‐1*, and *35:TPPE‐2* seedlings to medium supplemented with ABA and the antioxidant reduced glutathione (GSH) or the NADPH oxidase inhibitor diphenyleneiodonium chloride (DPI). Compared to the WT, *tppe‐1,* and *tppe‐2* seedlings were less sensitive to ABA, whereas *35:TPPE‐1* and *35:TPPE‐2* seedling showed increased sensitivity to 30 μM ABA in the medium. Moreover, the roots length were significantly greater in *35:TPPE‐1* and *35:TPPE‐2* plants grown in ABA medium containing GSH or DPI compared to these lines in ABA medium alone, indicating that the inhibition of root elongation by ABA was partially restored by treatment with a reducing reagent (Figure 5E). Together, these results suggest that TPPE is involved in ABA‐inhibited root elongation by modulating ROS accumulation.

## DISCUSSION

Trehalose is an essential molecule required for plant growth and development. This carbohydrate can also act as a stress‐protective agent to reduce damage to plant tissues and improve stress resistance (O'Hara et al. 2013; Delorge et al. 2014; Zhang et al. 2017). Although recent work showed that ABA and trehalose contents in plants increase under drought conditions (Lin et al. 2019), the relationship between trehalose and the ABA signaling pathway had been unclear. In the current study, we demonstrated that trehalose and ABA have synergistic effects on root growth and stomatal closure in *Arabidopsis*, implying that trehalose participates in the ABA signal transduction process. *TPPE*, the most ABA‐responsive gene in the *TPP* gene family, plays a key role in trehalose production. However, the mechanisms underlying the role of *TPPE* in ABA signaling in response to stress remain elusive. Here, we uncovered a possible regulatory network governing trehalose metabolism and ABA signaling to control root growth and stomatal movement.

We determined that TPPE plays an important role in ABA‐controlled root elongation and stomatal closure based on the following evidence. First, ABA induced trehalose accumulation in WT plants but not *tppe* mutants. The *tppe* mutants showed reduced sensitivity to ABA‐induced inhibition of root elongation and stomatal closure (Figure 2). Second, ABF2 directly binds to the promoter of *TPPE* and controls its expression (Figure 3). Third, overexpressing *ABF2* did not restore the phenotype of *tppe*, that is, its reduced sensitivity to ABA‐inhibited root elongation, implying that *TPPE* acts downstream of ABF2 (Figure 4). Taken together, these results indicate that ABA signaling modulates trehalose biosynthesis and that trehalose functions synergistically with ABA. Such a regulatory mechanism might help the plant respond rapidly to adverse conditions by balancing plant growth and defense responses.

Abscisic acid induces a rapid stomatal response to environmental stimuli by activating or repressing ion channels and other signaling components (Munemasa et al. 2015; Wang et al. 2019a). Our results indicate that ABA induces the expression of members of the *TPP* family to some extent. Interestingly, the expression levels of *TPPs* remained above basal levels 6 h after rapid upregulation, suggesting that relatively high levels of trehalose are associated with the long‐term effect of ABA on plant growth. Abscisic acid contents fluctuate in a rhythmic fashion, reaching their highest levels at 12 h after dawn (Adams et al. 2018; Belbin and Dodd 2018). The changes in trehalose contents follow the same rhythmical pattern, reaching maximum levels after 12 h of daylight (Carillo et al. 2013). Therefore, our results support the correlation between ABA levels and trehalose accumulation.

ABA RESPONSE ELEMENT BINDING FACTOR2, ABF3, and ABF4 are key transcription factors that function in various plant responses to ABA signals induced by cold and osmotic stress (Choi et al. 2000; Yoshida et al. 2010; Fujita et al. 2013; Yoshida et al. 2014). However, our study provided no evidence that ABF3 and ABF4 directly bind to the *TPPE* promoter. ABF3 and ABF4 play important roles in regulating seed germination and plant responses to ABA and various stresses, whereas ABF2 primarily functions in ABA responses during vegetative growth (Kim et al. 2004; Yoshida et al. 2015; Zandkarimi et al. 2015). *TPPE* expression and trehalose content were significantly lower in the *abf2* mutants compared to the WT (Figure 4B, C). In addition, our results demonstrate that trehalose content is controlled by ABF2 in an ABA‐dependent manner (Figure 4A), suggesting that core ABA signaling modulates trehalose metabolism. This idea is further supported by the finding that the trehalose content in *open stomata1* (*OST1*) mutants (which lack a kinase downstream of ABA perception) remained unchanged after ABA treatment (data not shown). However, some stress‐response genes function through an ABA‐independent pathway. Analysis of the promoters of these genes revealed that the dehydration response element (DRE; TACCGACAT) and C‐repeat (CRT) element (G/ACCGAC) are primarily activated independently of ABA (Baker et al. 1994; Yamaguchi‐Shinozaki and Shinozaki 1994). DREB1A combines with DRE/CRT at the *TPPF* promoter and activates its expression to increase soluble sugar contents, resulting in improved drought resistance (Lin et al. 2019). Although it remains unclear whether *TPPF* activity is independent of ABA, Lin demonstrated that trehalose content is regulated in many different manners in plants coping with different stresses. It remains to be elucidated whether these pathways are interrelated or integrated into a regulatory network.

Compared to other disaccharides, trehalose levels are very low in plants (Lunn et al. 2014). Transgenic plants expressing bacterial or plant TPS or TPP were previously generated in studies aiming to improve plant stress tolerance; however, although these plants showed drought tolerance, changes in trehalose contents were not obvious in the transgenic plants (Romero et al. 1997; Pramanik and Imai 2005; Li et al. 2011). Another study used constitutive or stress‐inducible promoters to express *TPS* genes and also induced drought tolerance (Karim et al. 2007). Trehalose is thought to function as a signaling molecule affecting sugar metabolism, cell wall modification, and ROS production (Chary et al. 2008; Van Houtte et al. 2013; Yang et al. 2014). Exogenous treatment with trehalose increases the cellular levels of superoxide anion (O_2_^−^) and hydrogen peroxide (H_2_O_2_) by upregulating the expression of NADPH oxidase genes *RBOHD/F* (Shi et al. 2019). In the current study, increasing endogenous trehalose contents induced ROS production. Glutathione and DPI treatment partially reduced the synergistic effects of ABA and trehalose on inhibiting root elongation (Figure 5E). Therefore, the effects of ABA and trehalose on the suppression of root elongation could be attributed, at least in part, to increased ROS production.

Trehalose induces stomatal closure via ROS accumulation (Shi et al. 2019), whereas ABA has little effect on stomatal movement in the *tppg* and *tps1* mutants (Gomez et al. 2010; Vandesteene et al. 2012). Here, we show that stomatal closure was less sensitive to ABA in the *tppe* mutants than in the WT (Figure 2C), suggesting that TPPE‐controlled trehalose plays an important role in stomatal closure. However, we also detected increased ROS accumulation in guard cells of *TPPE*‐overexpressing plants, but their stomatal response to ABA and the rate of water loss in detached leaves were similar to those of WT plants. These results suggest that overexpressing *TPPE* has antagonistic effects on ROS‐promoted stomatal closure and that stomatal movement is controlled by a complex regulatory network.

**Figure 6:**
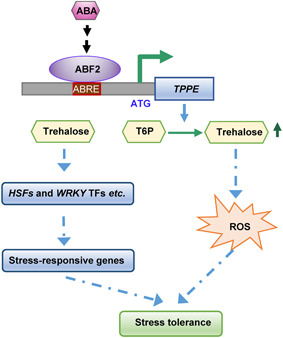
**Proposed model of the role of trehalose in abscisic acid (ABA)‐controlled root growth and stomatal responses** In the presence of ABA, ABA RESPONSE ELEMENT BINDING FACTOR2 (ABF2) enhances *TPPE* expression by directly binding to its promoter. The trehalose content increases due to enhanced *TPPE* expression. Trehalose triggers reactive oxygen species (ROS) accumulation in roots and stomata, which enhances the effect of ABA on inhibiting root growth and promoting stomatal closure, allowing the plant to respond rapidly to stress conditions. In addition, trehalose acts upstream of transcription factors to regulate stress‐responsive gene expression (Shi et al. 2019).

Based on our results, we propose a model in which a trehalose‐mediated branch of ABA signaling controls root growth (Figure 6). In the presence of ABA or under stress conditions, ABA signaling is activated. Consequently, ABF2, a key transcription factor that functions downstream of ABA signaling, activates the expression of *TPPE*, which leads to trehalose accumulation. Trehalose‐induced ROS accumulation enhances the effects of ABA. In the absence of ABA or when the stress conditions end, the plant returns to normal growth conditions, ABF2 loses its regulatory effect on *TPPE*, and *TPPE* expression and trehalose production return to basal levels to maintain normal plant growth and development. Our study provides a molecular basis for the role of trehalose in plants under abiotic stress and uncovers a new downstream regulatory branch of the ABA signaling pathway.

## MATERIALS AND METHODS

### Plant materials and growth conditions

The *A. thaliana* ecotype Columbia‐0 (Col‐0) was used as the WT control. T‐DNA insertion mutants *tppe‐1* (Salk_090223), *tppe‐2* (GK‐291G05), and *abf2* (Salk_002984) were obtained from ABRC (Arabidopsis Biological Resource Center). The seeds were surface sterilized for 15 min in 10% bleach, washed four times with sterile water, and plated on half‐strength Murashige and Skoog (½ MS) medium. The seeds were stratified at 4°C for 2 d in the dark and transferred to a phytotron at 22°C with a 16‐h light: 8‐h dark photoperiod (light intensity 100 μmol /(m^2^·s)). *N. benthamiana* was grown under a 16‐h light: 8‐h dark photoperiod. All experiments were repeated at least three times.

### Construction of plasmids and generation of transgenic plants

*TPPE*‐overexpressing lines *35S:TPPE‐1* and *35S:TPPE‐2* were generated by cloning the coding sequence of *TPPE* into the pCAMBIA1300 vector under the control of the CaMV *35S* promoter. The construct was used for transformation (via *Agrobacterium* strain GV3101) by the floral‐dip method. Transgenic seedlings were selected on ½ MS medium containing 25 mg/L hygromycin (Sigma‐Aldrich). We obtained single‐copy lines and used T_3_ seeds for subsequent experiments.

### Stomatal movement assay

Stomatal movement assays were performed as described previously (Dong et al. 2018). Four‐week‐old rosette leaves were harvested and epidermal strips were incubated in stomatal opening solution containing 50 mM KCl, 10 μM CaCl_2_, and 10 mM MES‐KOH (pH 6.1) for 2.5 h in the light, followed by the addition of 10 μM ABA and 2 h of incubation. Subsequently, the epidermal strips were mounted on glass slides, imaged under a Nikon 80i upright microscope, and measured using ImageJ software. The stomatal aperture values are means from at least 100 stomata, measured in five plants per treatment.

### RNA isolation and quantitative RT‐PCR

The *Arabidopsis* seedlings (100 mg) were harvested in liquid ½ MS medium (with or without 50 μM ABA). Total RNA was extracted from the samples using Trizol reagent (Invitrogen), and DNA contamination was removed by DNaseI (Invitrogen) treatment. First‐strand complementary DNA (cDNA) was synthesized with the Reverse Transcription System (Promega) and used as a template for qRT‐PCR with LightCycler 480 SYBR Green I Master (Roche). qRT‐PCR was performed on a Roche 480 Real‐Time PCR System following the manufacturer's instructions. Results were normalized to *ACTIN2/8*.

### Measuring trehalose content

To measure trehalose contents, 10‐d‐old *Arabidopsis* seedlings (100 mg) were harvested and rapidly frozen in liquid nitrogen. After being ground to powder, each sample was evenly mixed with 500 μL chloroform: acetonitrile (3:7, v‐v) and incubated in a ‐10°C freezer with occasional shaking. The organic matter was extracted with 400 μL water at 4°C and centrifuged for 4 min at 10,000 *g*; this step was repeated twice. The supernatants were combined and dried in a centrifugal vacuum dryer. The dry samples were dissolved in 500 μL methanol: water (1:1, v‐v) and filtered through a 0.22 μm membrane at room temperature.

ACQUITY UPLC I‐Class (Waters) coupled with a Xevo G2 Q‐TOF high‐resolution mass spectrometer (Waters) was used to measure trehalose content. Chromatographic separation was conducted using a UPLC BEH Amide column (1.7 μm, 2.1 mm × 100 mm, Waters). Elution was performed with mobile 90% phase A (water, 0.1% ammonia) and 10% phase B (acetonitrile: water = 95:5, 0.1% ammonia, 2 mM ammonium acetate) at a flow rate of 0.3 mL/min.

### Measuring ABA content

Ten‐d‐old seedlings (100 mg) were subjected to endogenous ABA measurements. After treatment with trehalose in ½ MS liquid medium for 4 h, the seedlings were harvested and immediately frozen in liquid nitrogen, then ground to powder. Extraction solvent (acetone, water, acetic acid, 80:19:1, v‐v:v) was added to the sample and carefully mixed. The supernatant was recovered by centrifugation; this step was repeated twice. The extraction solvent was evaporated and the residue was resuspended in 0.5 mL dissolution solvent (acetonitrile, water, 30:70, v‐v). Abscisic acid was quantified using an LC‐ESI‐MS‐MS system (Quattro LC, Waters) in negative ionization and multiple reaction‐monitoring mode.

### Yeast one‐hybrid assay

The yeast one‐hybrid (Y1H) assay was carried out using the Matchmaker Gold Yeast One‐Hybrid System (Clontech). The three bait fragments (*E1*, *E2*, and *E3* fragments) of the *TPPE* promoter were amplified by PCR and inserted into the pAbAi vector independently; this vector harbors the *AbA* ^*r*^ reporter gene. The constructs were linearized by *Bst*BI digestion and transformed into Y1H Gold cells. The full‐length coding sequences of the *ABF2* were cloned into the pGADT7 vector. The constructs were transformed into the Y1H bait strain and cultured on plates containing SD/‐Leu medium supplemented with 100 ng/mL Aureobasidin A (Clontech). The m*E3* fragment was synthesized and used as a negative control.

### Electrophoretic mobility shift assay

For EMSA, the full‐length cDNA of *ABF2* was amplified and cloned into the pMAL‐c5E vector (New England Biolabs). The recombinant MBP‐ABF2 protein was purified from *E. coli*. Oligonucleotides based on the *TPPE* promoter were synthesized and labeled with biotin at the 3′‐end. Unlabeled versions of the same oligonucleotides were used as competitors. The probes were obtained by annealing using biotin‐labeled or unlabeled primers. Electrophoretic mobility shift assay was performed using a LightShift Chemiluminescent EMSA kit (Thermo Fisher Scientific). Briefly, a 20 μL reaction mixture containing 2 μL of binding buffer, 0.5 μL of poly (dI‐dC), 1 μg of purified fusion protein, and 1 μL of biotin‐labeled probe was incubated at room temperature for 20 min. For competition with unlabeled probes, unlabeled probes were added to the reactions, which were incubated for 20 min. The reactions mixture were resolved in 5% native polyacrylamide gels with 1 × TBE buffer. The assays were repeated three times.

### Chromatin immunoprecipitation assay

Chromatin immunoprecipitation assay was performed as described previously (Wang et al. 2019a). Two‐week‐old *35S:ABF2‐GFP* transgenic plants and 3*5S:GFP* plants grown on ½ MS medium (5 g) were collected for chromatin extraction using a ChIP‐A kit (Millipore). The isolated chromatin was sonicated to produce 0.5–1.0 kb DNA fragments. The DNA fragments were purified and resuspended in double‐distilled water, and enriched DNA fragments were quantified by qPCR. Fold enrichment of each region in the *35S:ABF2‐GFP* line was calculated compared to that in the *35S:GFP* line.

### Transient expression assays in *Nicotiana benthamiana*

The full‐length cDNA of *ABF2* was inserted into the pCAMBIA1300 vector under the control of the *35S* promoter, and the *TPPE* promoter fragment was cloned into the pCAMBIA1300‐*LUC* vector. For transient expression, plasmids were transferred into *Agrobacterium* strain GV3101. *Nicotiana benthamiana* plants were grown for 5‐6 weeks in a growth chamber. For infiltration, *Agrobacterium* cultures were grown overnight, collected by centrifugation (4,000 *g*), and resuspended in infiltration medium (10 mM MES, pH 5.6, 10 mM MgCl_2_ and 200 μM acetosyringone) to a final optical density at 600 nm (OD_600_) of 0.3. The suspensions were mixed and incubated for 2 h at room temperature on a horizontal rolling mixer. Co‐infiltration into the abaxial surfaces of *N. benthamiana* leaves was performed using a 1 mL needle‐free syringe. The infiltrated plants were incubated under a 10‐h light: 14‐h dark photoperiod at 22°C for 48 h to express LUC proteins. The LUC luminescence intensity of the infiltrated leaves was observed by bioluminescence imaging. In each experiment, 10 independent *N. benthamiana* leaves were infiltrated and analyzed.

### Protoplast transformation and dual‐luciferase reporter assay

Protoplasts were isolated from the rosette leaves of 4‐week‐old WT plants as described (Yoo et al. 2007). pCAMBIA1300‐*LUC* vector containing four tandem copies of the ABRE‐containing fragment was used as reporter, and the *35S:ABF2‐GFP* construct was used as an effector. The constructs were co‐transfected into protoplasts by polyethylene glycol‐mediated transfection. For ABA treatment, protoplasts were incubated overnight in 10 μM ABA after transfection. Firefly and Renilla luciferase activities were quantified using a dual‐luciferase assay kit (Promega) and detected using a Synergy 2 multi‐mode microplate reader (Bio‐Tek).

### Measuring ROS in plants

Four‐d‐old WT, *tppe‐1*, *tppe‐2*, *35S:TPPE‐1*, and *35S:TPPE‐2* seedlings were incubated in liquid ½ MS with or without 50 μM ABA for 4 h before staining. For NBT staining to detect superoxides, the seedlings were incubated in buffer containing 1 mM NBT (Sigma‐Aldrich), 20 mM K‐phosphate, and 0.1 M NaCl at pH 6.2 for 15 min. The seedlings were cleared by washing three times with water, transferred to a solution containing 7% NaOH and 60% ethanol, and incubated for 15 min at room temperature. The seedlings were further incubated for 10 min at 90°C in the following ethanol series: 50% ethanol, 70% ethanol, and 90% ethanol. The seedlings were examined under an Olympus BX53 microscope. For 2′, 7′‐dichlorodihydrofluorescein diacetate (H_2_DCFDA) staining to detect H_2_O_2_, the seedlings were incubated in buffer containing 5 μM H_2_DCFDA (Sigma‐Aldrich) and 20 mM PBS in the dark for 10 min. The roots were photographed under a Zeiss LSM510 META confocal microscope with excitation at 488 nm. The intensities of the fluorescent signals were statistically compared using Student's *t*‐test.

## AUTHOR CONTRIBUTIONS

C.‐P.S. and X.Z. conceived and designed research; W.W., Q.C., and S.X. performed the experiments; W.‐C.L. interpreted and analyzed the results; C.‐P. S., W.W., and X.Z. analyzed the data and wrote the manuscript.

## COMPETING FINANCIAL INTERESTS

The authors declare no competing financial interests.

## Supporting information

Additional Supporting Information may be found online in the supporting information tab for this article: http://onlinelibrary.wiley.com/doi/10.1111/jipb.12925/suppinfo

Supporting information.

**Figure S1.**
Expression levels of the genes in ABA biosynthetic pathway in the presence of exogenous trehalose
Ten‐d‐old WT seedlings grown on ½ MS medium were transferred to ½ MS liquid medium with or without trehalose (10 mM, 20 mM) for 12 h, and gene transcripts were analyzed by qRT‐PCR. Values show average ± SD (*n* = 3).

**Figure S2.**
Relative expression of *TPP*s induced by ABA
qRT‐PCR analysis reveals that *TPPs* expression is induced by ABA. Ten‐d‐old seedlings were treated with 50 μM ABA and collected for RNA extraction. *Actin2/8* was used as an internal standard. Values are the mean ± *SD* of three independent biological replicates.

**Figure S3.**
Histochemical analysis of *TPPE* promoter activity under ABA treatments
**(A)** Ten‐d‐old *proTPPE:GUS* transgenic seedlings were treated by ABA (50 μM) for 4 h and then harvested for GUS staining. Scale bar, 1 mm. **(B)** Quantitative analysis of GUS activity in *proTPPE:GUS* transgenic seedlings under ABA treatments. Values are mean ± *SD* of three replicate experiments (Student's *t‐*test, **P < 0.01).

**Figure S4.**
Analysis of TPPE enzymatic catalytic activity
**(A)** Standard curve of phosphate derived from the reaction of catalytic activity of TPPE. **(B)** Comparison of TPPE catalytic activities with different substrates.

**Figure S5.**
Identification of *tppe* mutant and *35S:TPPE* transgenic lines
**(A)** Schematic diagram of *TPPE* T‐DNA insertion lines. Black boxes are exons and lines between the boxes are introns. ATG and TGA are the start codon and termination codon, respectively. The position of the T‐DNA insertion is indicated by a triangle. **(B)** PCR analysis of the *tppe* insertion mutants. The genomic DNA products were PCR‐amplified using primer pairs LP + RP, LP + LBa1. **(C)** qRT‐PCR analysis of *TPPE* transcript levels in *tppe* mutants. **(D)** qRT‐PCR analysis of *TPPE* transcript levels in *35S:TPPE* lines. Ten‐d‐old seedlings were used for qRT‐PCR analysis.

**Figure S6.**
The generation and phenotype analysis of the *TPPE* CRISPR/Cas9 mutants
**(A)** The schematic map of the gRNA targeted sites of *TPPE*. **(B)** The sequencing chromatograms show the positions of the deletion in *tppe‐cas9‐1* and *tppe‐cas9‐2* mutants. **(C)** Phenotypic analysis of WT and *tppe‐cas9* lines under ABA treatment. Scale bar, 1 cm. **(D)** Statistical analysis of the root length corresponding to **(C)**. Error bars indicate ± *SD* (*n* = 9), *P < 0.05, **P < 0.01. **(E)** Water loss from the detached leaves of WT, *tppe‐cas9‐1* and *tppe‐cas9‐2*. The experiments were repeated three times with similar results. Each data point represents the means ± SD (*n* = 3).

**Figure S7.**
Phenotype analysis of *abf2* mutant and *ABF2* overexpression lines
**(A)** Schematic diagram of T‐DNA insertion lines of *abf2*. Black boxes are exons, and lines between the boxes are introns. ATG and TGA are the start codon and termination codon. **(B)** Identification of *abf2* mutants by PCR. The genomic DNA products were PCR‐amplified using primer pairs LP + RP, LP + LBa1. **(C)** qRT‐PCR analysis of the expression of *ABF2* in mutant and overexpression lines. **(D)** The root length of mutants and overexpression lines under ABA treatment. The values are means ± *SD* (*n* > 10). Different letters indicate statistical differences at *P* < 0.05 (one‐way ANOVA). **(E)** The expression of *TPPE* and *ABF2* in double mutants. The gene expression was detected by qRT‐PCR.

**Figure S8.**
ABA induces ROS production in the guard cells of WT, *tppe‐1, tppe‐2*, *35S:TPPE‐1* and *35S:TPPE‐2* plants
**(A)** H_2_DCFDA staining for ROS in guard cells, Scale bar, 10 μm. **(B)** The intensity of the fluorescence signal was measured by Image J. The values are means ± *SD* (*n* > 10). Different letters indicate statistical differences at *P* < 0.05 (one‐way ANOVA).
