# Supplementary material for: Trehalose‐6‐phosphate phosphatase E modulates ABA‐controlled root growth and stomatal movement in Arabidopsis
Source: J Integr Plant Biol. 2020 Apr 16;62(10):1518–34. doi: 10.1111/jipb.12925 (PMC7586804; doi:10.1111/jipb.12925)
Supplement: Supplementary file 1 — Supporting information. [file JIPB-62-1518-s001.docx]

**SUPPLEMENTARY METHODS**

***proTPPE:GUS* construct and GUS activity**

To generate the *proTPPE:GUS* transgenic lines, a 2000 bp DNA fragment from the region upstream from the *TPPE* coding sequence was cloned into the pCAMBIA1381 vector. For GUS staining, the transgenic seedlings with or without ABA treatment were immersed in a staining solution (100 mM sodium-phosphate buffer, pH 7.0, 1 mM K_4_Fe(CN)_6_, 1 mM K_3_Fe(CN)_6_, 0.1% Triton X-100, 2 mM X-Gluc) for 6 h at 37°C in the dark and followed by two times washes with 70% ethanol to remove chlorophyll. Samples were photographed using a stereoscope (Leica) equipped with a CCD camera. To test for GUS expression before and after ABA, seedlings were treated with 50 μM ABA for 4 h.

**Generation and screening of CRISPR/Cas9 edited lines**

The CRISPR-Cas9 cloning plasmids pHSN401 was used in this study. A 23 nt (5'-N20 NGG-3') target sites was selected from the exonic regions of *TPPE* based on the *Arabidopsis* *thaliana* genomic DNA database (http://www.arabidopsis.org/), and the Cas-OFFinder database was searched for highly specific target site sequences (http://www.rgenome.net/cas-offinder/). The pHSN401 vector was digested with *Bsa*I restriction enzyme (New England Biolabs) and was ligated with gRNA by using T4 DNA fast ligases (New England Biolabs). The *E.coli* cells were transformed with the ligation product, and the recombinant was selected by picking a single clone and sequencing. The obtained positive recombinant construct was introduced into WT by the *Agrobacterium* strain GV3101 mediated floral infiltration method. The transgenic seedlings were selected on ½ MS medium containing 25 mg/L hygromycin (Sigma-Aldrich) and their genomic DNA was extracted using the CTAB method. The primers with flanking regions were designed to amplify the target sites, and sequencing screen the positive mutants.

***In* *vitro* phosphatase activity of TPPE**

The phosphatase activity of TPPE *in* *vitr*o was performed by the Malachite Green Phosphate Assay Kit (Sigma-Aldrich) following the manufacturer’s instructions. Briefly, add 0, 10, 20, 40, 80 μL of 1 mM Phosphate Standard and 30 μL Working Reagent into individual wells. Adjust the volume to 200 μL with ddH_2_O and incubate at room temperature for 30 min to detect the absorbance at 650 nm and generate a standard curve for quantification of the reaction product. To get the activity of TPPE, 2 μg TPPE-His expressed in *E.coli* was added to 5× enzymatic reaction buffer into the well with 10 μL T6P, S6P, G6P (10 mM) at 30°C for 3 hours, respectively. Add 30 μL Working Reagent into each pore and incubate at room temperature for 30 min after reaction. Detect the absorbance at 650 nm, and calculate the concentration of free phosphate according to the standard curve.

**FIGURE LEGEND**

**Figure S1. Expression levels of the genes in ABA biosynthetic pathway in the presence of exogenous trehalose.** Ten-day-old WT seedlings grown on ½ MS medium were transferred to ½ MS liquid medium with or without trehalose (10 mM, 20 mM) for 12 h, and gene transcripts were analyzed by qRT-PCR. Values show average ± SD (*n* = 3).

**Figure S2. Relative expression of *TPP*s induced by ABA.** qRT-PCR analysis reveals that *TPPs* expression is induced by ABA. Ten-day-old seedlings were treated with 50 μM ABA and collected for RNA extraction. *Actin2/8* was used as an internal standard. Values are the mean ± SD of three independent biological replicates.

**Figure S3. Histochemical analysis of *TPPE* promoter activity under ABA treatments. (A)** Ten-day-old *proTPPE:GUS* transgenic seedlings were treated by ABA (50 μM) for 4 h and then harvested for GUS staining. Scale bar, 100 μm. **(B)** Quantitative analysis of GUS activity in *proTPPE:GUS* transgenic seedlings under ABA treatments. Values are mean ± SD of three replicate experiments (Student’s *t-*test, ** *P* < 0.01).

**Figure S4. Analysis of TPPE enzymatic catalytic activity. (A)** Standard curve of phosphate derived from the reaction of catalytic activity of TPPE. **(B)** Comparison of TPPE catalytic activities with different substrates.

**Figure S5.** **Identification of *tppe* mutant and *35S:TPPE* transgenic lines**. **(A)** Schematic diagram of *TPPE* T-DNA insertion lines. Black boxes are exons and lines between the boxes are introns. ATG and TGA are the start codon and termination codon, respectively. The position of the T-DNA insertion is indicated by a triangle. **(B)** PCR analysis of the *tppe* insertion mutants. The genomic DNA products were PCR-amplified using primer pairs LP + RP, LP + LBa1. **(C)** qRT-PCR analysis of *TPPE* transcript levels in *tppe* mutants. **(D)** qRT-PCR analysis of *TPPE* transcript levels in *35S:TPPE* lines. Ten-day-old seedlings were used for qRT-PCR analysis.

**Figure S6. The Generation and phenotype analysis of the *TPPE* CRISPR/Cas9 mutants. (A)** The schematic map of the gRNA targeted sites of *TPPE*. **(B)** The sequencing chromatograms show the positions of the deletion in *tppe-cas9-1* and *tppe-cas9-2* mutants. **(C)** Phenotypic analysis of WT and *tppe-cas9* lines under ABA treatment. Scale bar, 1 cm. **(D)** Statistical analysis of the root length corresponding to (C). Error bars indicate ± SD (*n* = 9), * *P* < 0.05, ** *P* < 0.01. **(E)** Water loss from the detached leaves of WT, *tppe-cas9-1* and *tppe-cas9-2*. The experiments were repeated three times with similar results. Each data point represents the means ± SD (*n* = 3).

**Figure S7. Phenotype analysis of *abf2* mutant and *ABF2* overexpression lines. (A)** Schematic diagram of T-DNA insertion lines of *abf2*. Black boxes are exons, and lines between the boxes are introns. ATG and TGA are the start codon and termination codon. **(B)** Identification of *abf2* mutants by PCR. The genomic DNA products were PCR-amplified using primer pairs LP + RP, LP + LBa1. **(C)** qRT-PCR analysis of the expression of *ABF2* in mutant and overexpression lines. **(D)** The root length of mutants and overexpression lines under ABA treatment. The values are means ± SD (*n* > 10). Different letters indicate statistical differences at *P* < 0.05 (one-way ANOVA analysis). **(E)** The expression of *TPPE* and *ABF2* in double mutants. The gene expression was detected by qRT-PCR.

**Figure S8**. **ABA induces ROS production in the guard cells of WT, *tppe-1, tppe-2*, *35S:TPPE-1* and *35S:TPPE-2* plants*.* (A**) H_2_DCFDA staining for ROS in guard cells, Scale bar, 10 μm. **(B)** The intensity of the fluorescence signal was measured by Image J. The values are means ± SD (*n* > 10). Different letters indicate statistical differences at *P* < 0.05 (one-way ANOVA analysis).
