# Supplementary figures and images for: Trehalose‐6‐phosphate phosphatase E modulates ABA‐controlled root growth and stomatal movement in Arabidopsis
Source: J Integr Plant Biol. 2020 Apr 16;62(10):1518–34. doi: 10.1111/jipb.12925 (PMC7586804; doi:10.1111/jipb.12925)

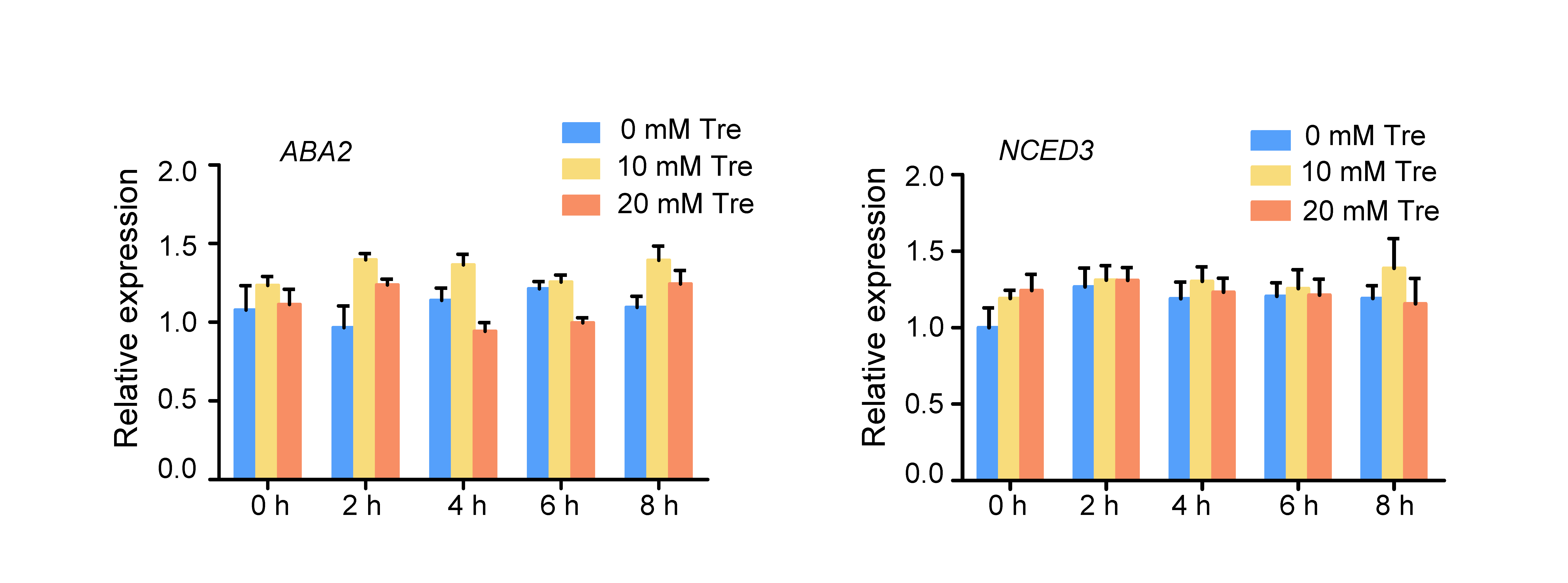

Supplement: Supplementary file 2 — Figure S1. Expression levels of the genes in ABA biosynthetic pathway in the presence of exogenous trehalose Ten‐d‐old WT seedlings grown on ½ MS medium were transferred to ½ MS liquid medium with or without trehalose (10 mM, 20 mM) for 12 h, and gene transcripts were analyzed by qRT‐PCR. Values show average ± SD (n = 3). [file JIPB-62-1518-s002.jpg]

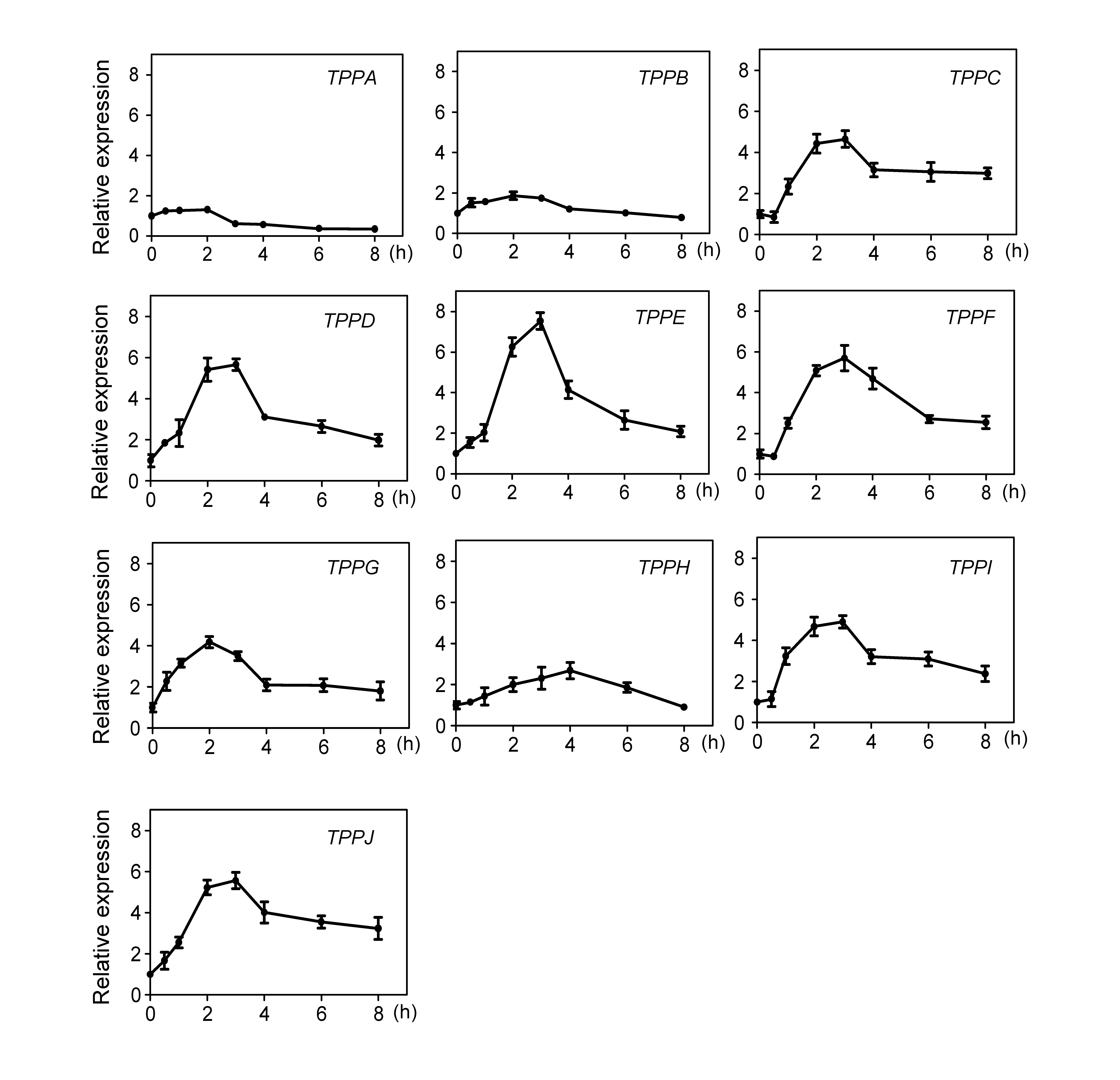

Supplement: Supplementary file 3 — Figure S2. Relative expression of TPPs induced by ABA qRT‐PCR analysis reveals that TPPs expression is induced by ABA. Ten‐d‐old seedlings were treated with 50 μM ABA and collected for RNA extraction. Actin2/8 was used as an internal standard. Values are the mean ± SD of three independent biological replicates. [file JIPB-62-1518-s003.jpg]

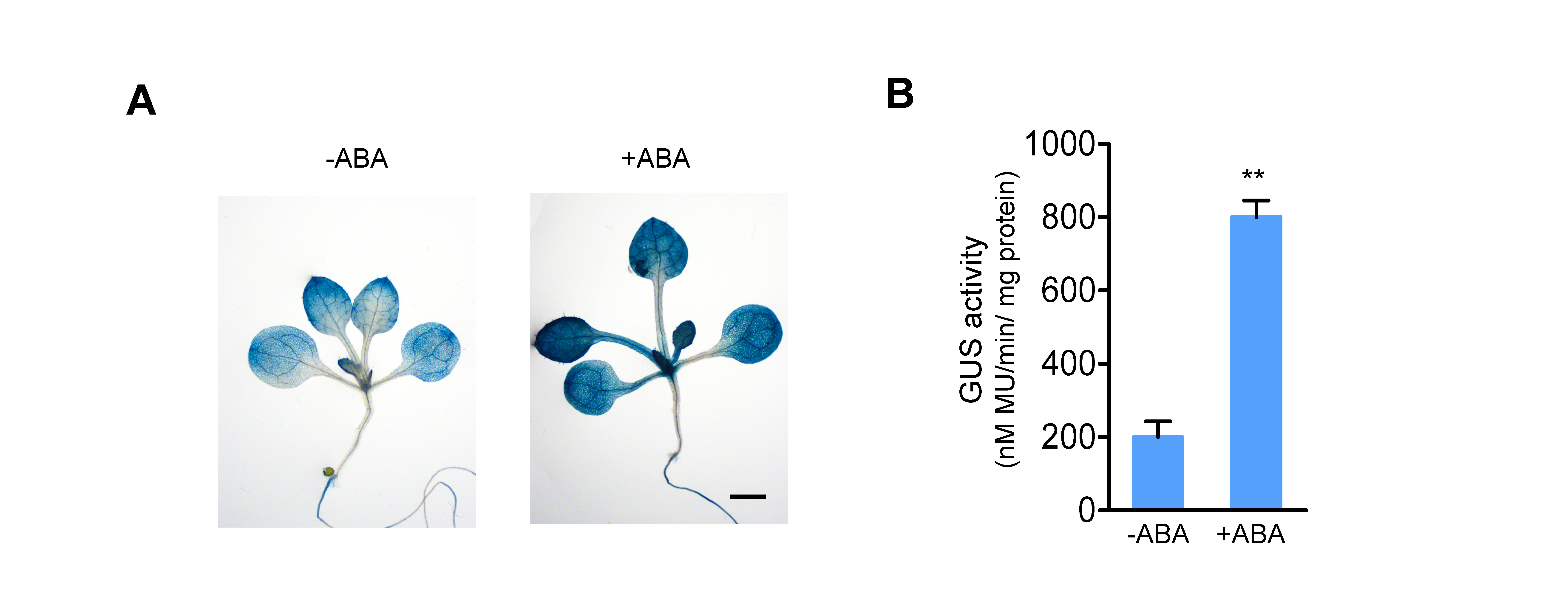

Supplement: Supplementary file 4 — Figure S3. Histochemical analysis of TPPE promoter activity under ABA treatments (A) Ten‐d‐old proTPPE:GUS transgenic seedlings were treated by ABA (50 μM) for 4 h and then harvested for GUS staining. Scale bar, 1 mm. (B) Quantitative analysis of GUS activity in proTPPE:GUS transgenic seedlings under ABA treatments. Values are mean ± SD of three replicate experiments (Student's t‐test, **P < 0.01). [file JIPB-62-1518-s004.jpg]

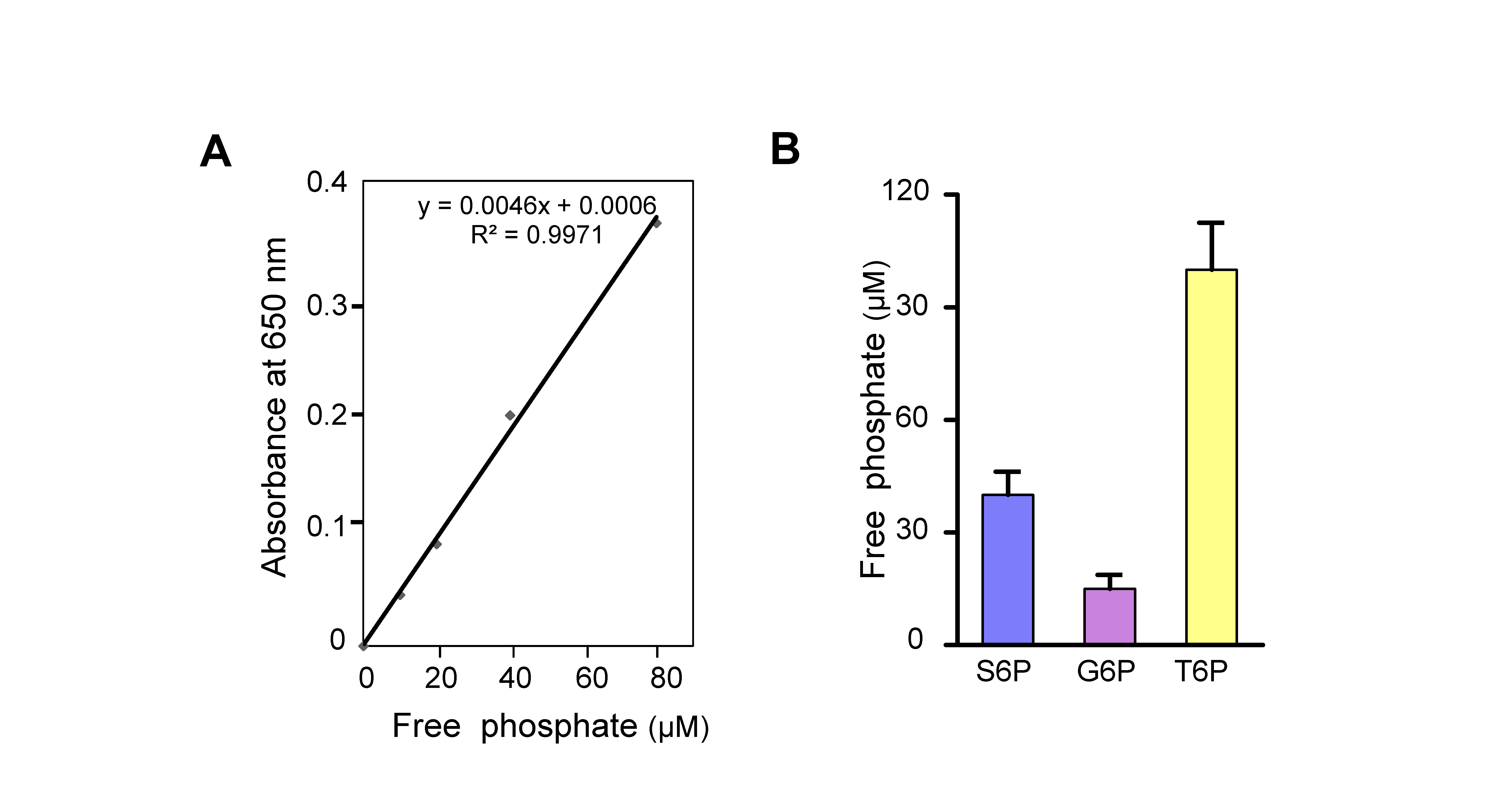

Supplement: Supplementary file 5 — Figure S4. Analysis of TPPE enzymatic catalytic activity (A) Standard curve of phosphate derived from the reaction of catalytic activity of TPPE. (B) Comparison of TPPE catalytic activities with different substrates. [file JIPB-62-1518-s005.jpg]

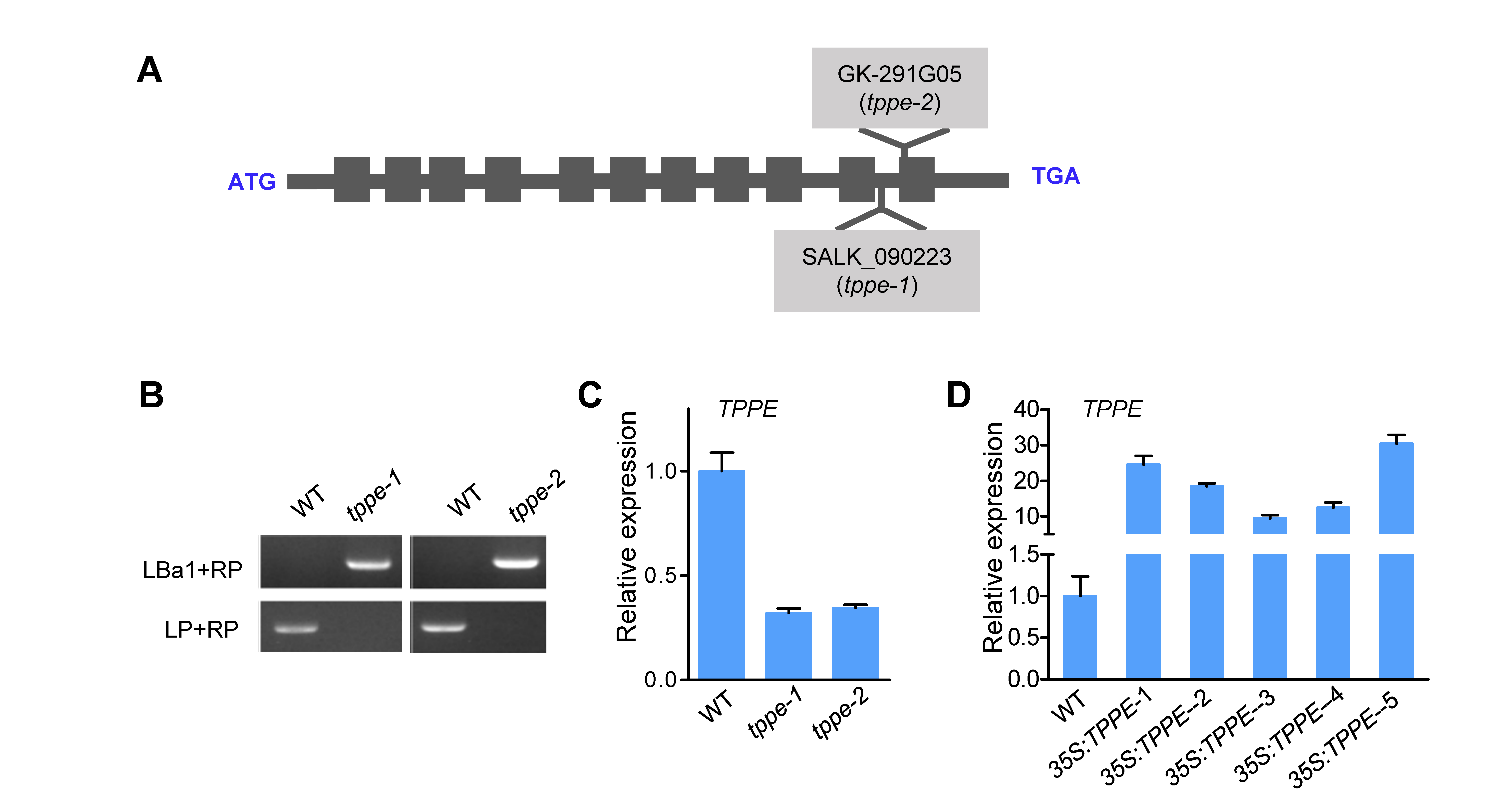

Supplement: Supplementary file 6 — Figure S5. Identification of tppe mutant and 35S:TPPE transgenic lines (A) Schematic diagram of TPPE T‐DNA insertion lines. Black boxes are exons and lines between the boxes are introns. ATG and TGA are the start codon and termination codon, respectively. The position of the T‐DNA insertion is indicated by a triangle. (B) PCR analysis of the tppe insertion mutants. The genomic DNA products were PCR‐amplified using primer pairs LP + RP, LP + LBa1. (C) qRT‐PCR analysis of TPPE transcript levels in tppe mutants. (D) qRT‐PCR analysis of TPPE transcript levels in 35S:TPPE lines. Ten‐d‐old seedlings were used for qRT‐PCR analysis. [file JIPB-62-1518-s006.jpg]

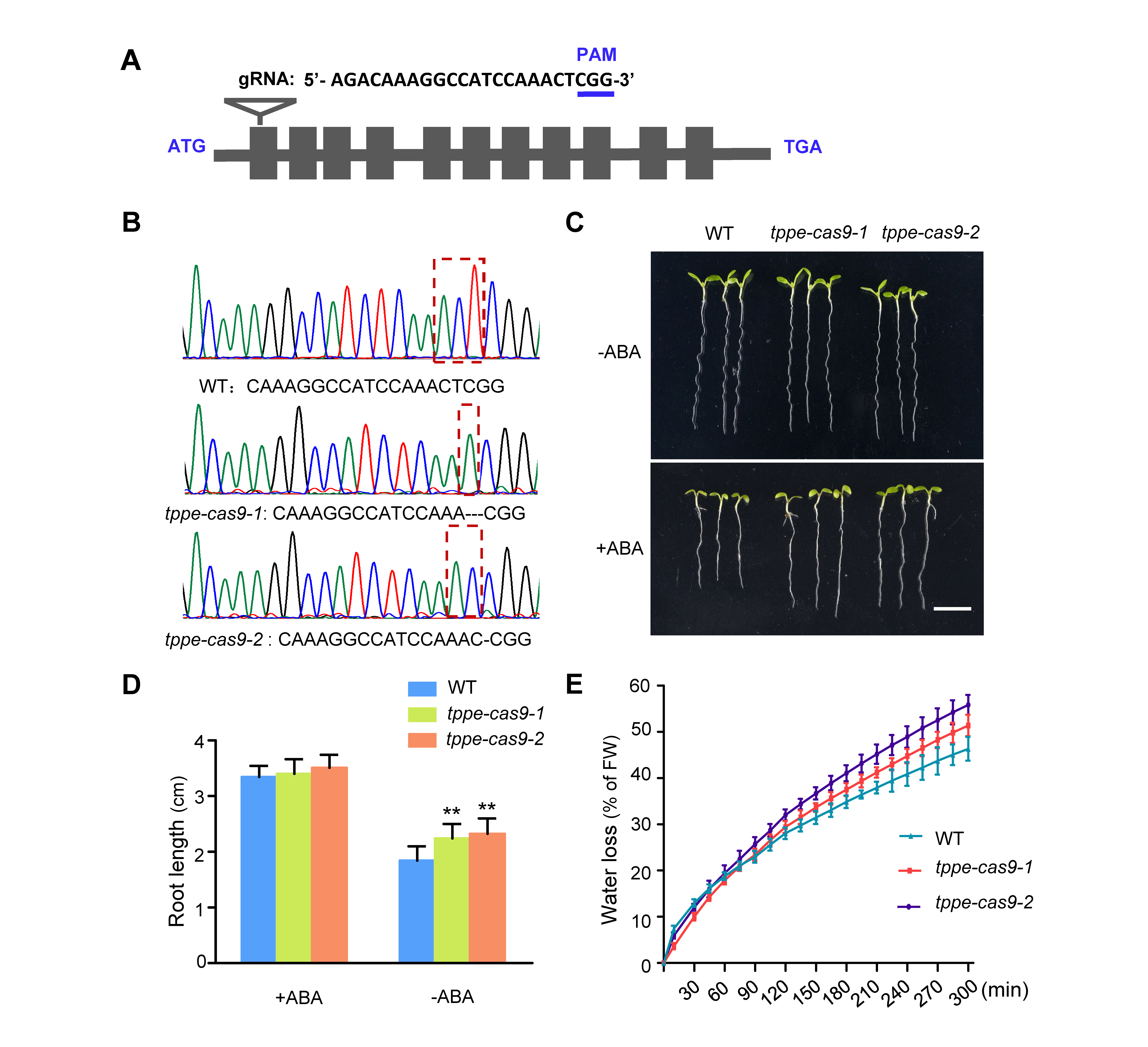

Supplement: Supplementary file 7 — Figure S6. The generation and phenotype analysis of the TPPE CRISPR/Cas9 mutants (A) The schematic map of the gRNA targeted sites of TPPE. (B) The sequencing chromatograms show the positions of the deletion in tppe‐cas9‐1 and tppe‐cas9‐2 mutants. (C) Phenotypic analysis of WT and tppe‐cas9 lines under ABA treatment. Scale bar, 1 cm. (D) Statistical analysis of the root length corresponding to (C). Error bars indicate ± SD (n = 9), *P < 0.05, **P < 0.01. (E) Water loss from the detached leaves of WT, tppe‐cas9‐1 and tppe‐cas9‐2. The experiments were repeated three times with similar results. Each data point represents the means ± SD (n = 3). [file JIPB-62-1518-s007.jpg]

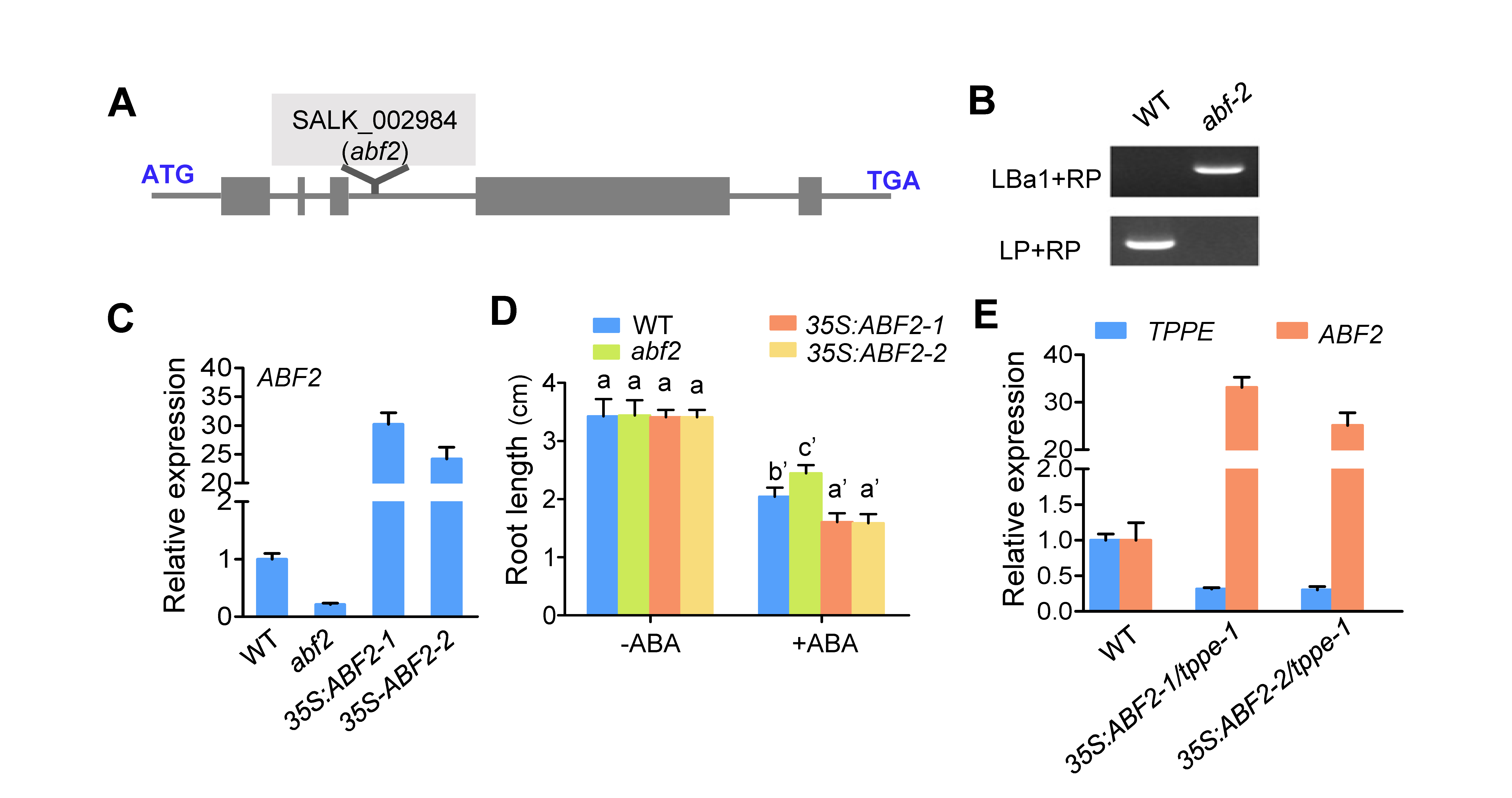

Supplement: Supplementary file 8 — Figure S7. Phenotype analysis of abf2 mutant and ABF2 overexpression lines (A) Schematic diagram of T‐DNA insertion lines of abf2. Black boxes are exons, and lines between the boxes are introns. ATG and TGA are the start codon and termination codon. (B) Identification of abf2 mutants by PCR. The genomic DNA products were PCR‐amplified using primer pairs LP + RP, LP + LBa1. (C) qRT‐PCR analysis of the expression of ABF2 in mutant and overexpression lines. (D) The root length of mutants and overexpression lines under ABA treatment. The values are means ± SD (n > 10). Different letters indicate statistical differences at P < 0.05 (one‐way ANOVA). (E) The expression of TPPE and ABF2 in double mutants. The gene expression was detected by qRT‐PCR. [file JIPB-62-1518-s008.jpg]

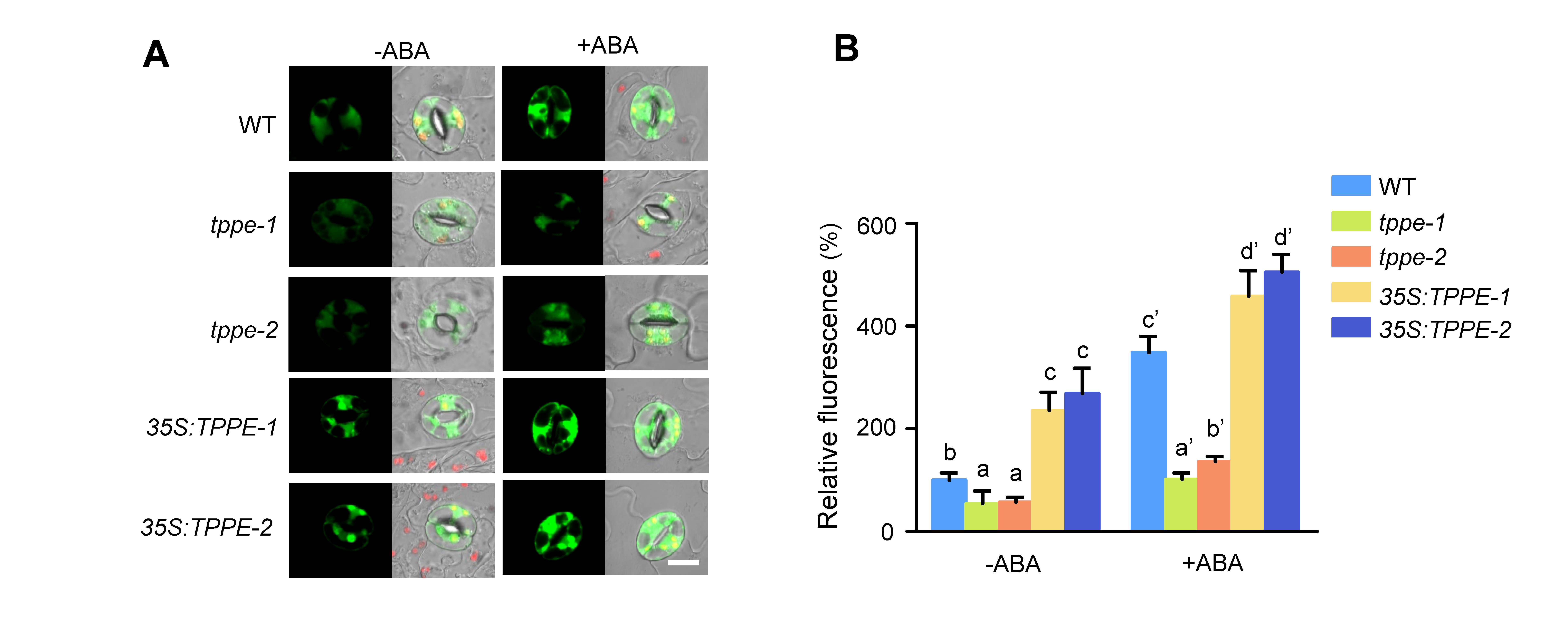

Supplement: Supplementary file 9 — Figure S8. ABA induces ROS production in the guard cells of WT, tppe‐1, tppe‐2, 35S:TPPE‐1 and 35S:TPPE‐2 plants (A) H2DCFDA staining for ROS in guard cells, Scale bar, 10 μm. (B) The intensity of the fluorescence signal was measured by Image J. The values are means ± SD (n > 10). Different letters indicate statistical differences at P < 0.05 (one‐way ANOVA). [file JIPB-62-1518-s009.jpg]
